# Phosphatidylserine-Liposomes Promote Tolerogenic Features on Dendritic Cells in Human Type 1 Diabetes by Apoptotic Mimicry

**DOI:** 10.3389/fimmu.2018.00253

**Published:** 2018-02-14

**Authors:** Silvia Rodriguez-Fernandez, Irma Pujol-Autonell, Ferran Brianso, David Perna-Barrull, Mary Cano-Sarabia, Sonia Garcia-Jimeno, Adrian Villalba, Alex Sanchez, Eva Aguilera, Federico Vazquez, Joan Verdaguer, Daniel Maspoch, Marta Vives-Pi

**Affiliations:** ^1^Immunology Section, Germans Trias i Pujol Research Institute, Autonomous University of Barcelona, Badalona, Spain; ^2^Statistics and Bioinformatics Unit, Vall d’Hebron Research Institute, Barcelona, Spain; ^3^Department of Genetics, Microbiology and Statistics, University of Barcelona, Barcelona, Spain; ^4^Catalan Institute of Nanoscience and Nanotechnology, CSIC and The Barcelona Institute of Science and Technology, Bellaterra, Spain; ^5^Endocrinology Section, Germans Trias i Pujol University Hospital, Badalona, Spain; ^6^Department of Experimental Medicine, University of Lleida & IRBLleida, Lleida, Spain; ^7^CIBERDEM, ISCiii, Madrid, Spain; ^8^ICREA, Barcelona, Spain

**Keywords:** immunotherapy, autoimmunity, human type 1 diabetes, liposomes, tolerance, dendritic cells

## Abstract

Type 1 diabetes (T1D) is a metabolic disease caused by the autoimmune destruction of insulin-producing β-cells. With its incidence increasing worldwide, to find a safe approach to permanently cease autoimmunity and allow β-cell recovery has become vital. Relying on the inherent ability of apoptotic cells to induce immunological tolerance, we demonstrated that liposomes mimicking apoptotic β-cells arrested autoimmunity to β-cells and prevented experimental T1D through tolerogenic dendritic cell (DC) generation. These liposomes contained phosphatidylserine (PS)—the main signal of the apoptotic cell membrane—and β-cell autoantigens. To move toward a clinical application, PS-liposomes with optimum size and composition for phagocytosis were loaded with human insulin peptides and tested on DCs from patients with T1D and control age-related subjects. PS accelerated phagocytosis of liposomes with a dynamic typical of apoptotic cell clearance, preserving DCs viability. After PS-liposomes phagocytosis, the expression pattern of molecules involved in efferocytosis, antigen presentation, immunoregulation, and activation in DCs concurred with a tolerogenic functionality, both in patients and control subjects. Furthermore, DCs exposed to PS-liposomes displayed decreased ability to stimulate autologous T cell proliferation. Moreover, transcriptional changes in DCs from patients with T1D after PS-liposomes phagocytosis pointed to an immunoregulatory prolife. Bioinformatics analysis showed 233 differentially expressed genes. Genes involved in antigen presentation were downregulated, whereas genes pertaining to tolerogenic/anti-inflammatory pathways were mostly upregulated. In conclusion, PS-liposomes phagocytosis mimics efferocytosis and leads to phenotypic and functional changes in human DCs, which are accountable for tolerance induction. The herein reported results reinforce the potential of this novel immunotherapy to re-establish immunological tolerance, opening the door to new therapeutic approaches in the field of autoimmunity.

## Introduction

Type 1 diabetes (T1D) mellitus is a metabolic disease caused by loss of tolerance to self and consequent autoimmune destruction of insulin-producing pancreatic β-cells (1). When β-cell mass decreases significantly, the individual’s endogenous production of insulin is no longer able to meet metabolic demands, leading to overt hyperglycemia. Upon diagnosis, patients with T1D require exogenous insulin administration, and although this treatment has allowed them to survive, long-term complications due to glycemic imbalances are bound to arise (2, 3). T1D usually appears during childhood or adolescence, and its incidence is increasing an average of 4% per year (4). Despite knowing that both genetic and environmental factors contribute to its development, triggering events remain elusive. The autoimmune attack against β-cells is led by a mild leukocytic infiltrate—insulitis—consisting of dendritic cells (DCs), macrophages, T and B lymphocytes, and natural killer cells, which gradually advance through the islets (5). The first islet-infiltrating cells are DCs (6), which orchestrate the loss of tolerance to β-cell autoantigens, insulin being a key autoantigen in human T1D (7).

The first step to revert T1D would be to arrest the pathological recognition of β-cell autoantigens. Many immunotherapies have prevented and even reverted T1D in animal models (8), but clinical trials have corroborated how challenging T1D prevention and reversion is, and most of them have been unsuccessful (9). In this scenario, the development of new therapies to halt autoimmunity in T1D has become an urgent biomedical matter. An ideal immunotherapy should restore tolerance to β-cells, avoiding systemic side effects, and allow islet regeneration. One of the most efficient physiological mechanisms for inducing tolerance is apoptosis, a form of programmed cell death lacking inflammation. The uptake of apoptotic cells by professional phagocytes such as macrophages and immature DCs (iDCs) is named efferocytosis (10). The exposure of “*eat-me*” signals on the apoptotic cell surface is what promotes their specific recognition and phagocytosis. Phosphatidylserine (PS), a phospholipid usually kept in the inner leaflet of the plasma membrane, is a relevant signal for efferocytosis. This molecule is recognized by multiple distinct receptors on antigen presenting cells, including members of the TIM family, Stabilin-2, integrins, CD36, CD68, among others, as well as by soluble receptors that in turn bind to membrane receptors (11). After the capture, the apoptotic cell is processed, thus prompting the release of anti-inflammatory signals and presentation of autoantigens in a tolerogenic manner by DCs (12). Failure of this mechanism, owing to an increase of apoptotic β-cells or defects in efferocytosis, contributes to the loss of tolerance to self in the context of T1D (13).

Our group demonstrated that DCs acquired a tolerogenic phenotype and functionality after engulfment of apoptotic β-cells, and that they prevented T1D when transferred to non-obese diabetic (NOD) mice (14, 15). However, since finding a substantial source of autologous apoptotic β-cells for T1D immunotherapy would be impossible, we conceived an immunotherapy based on biomimicry that consisted of liposomes—phospholipid bilayer vesicles—displaying PS in their surface and containing autoantigenic peptides, thus resembling apoptotic cells. Indeed, apoptotic mimicry performed by PS-liposomes successfully restored tolerance to β-cells in experimental autoimmune diabetes, preventing disease development and decreasing the severity of insulitis (16). Moreover, by only replacing the autoantigenic peptide encapsulated within PS-liposomes, we confirmed the potential of this immunotherapy to prevent and ameliorate experimental autoimmune encephalomyelitis, the experimental model of multiple sclerosis (17). In both cases, we demonstrated that phagocytosis of autoantigen-loaded PS-liposomes induced a tolerogenic phenotype and functionality in DCs, expansion of regulatory T cells and release of anti-inflammatory mediators that are responsible for arresting the autoimmune attack to target cells. Therefore, PS-liposomes could constitute a platform serving as a physiological and safe strategy to restore peripheral tolerance in antigen-specific autoimmune diseases. Liposomes, already used clinically as drugs deliverers for antitumor drugs and as vaccines (18), have the advantage of being safe and biocompatible, customizable, easily large-scale produced, and standardizable.

Aiming for the clinical potential of this strategy, we have encapsulated human insulin peptides to assess the effect of PS-liposomes in human DCs from patients with T1D and control subjects *in vitro*. We herein report that PS-liposomes are efficiently captured by human DCs, thus eliciting transcriptomic, phenotypic, and functional changes that point to tolerogenic potential. This immunotherapy constitutes a promising strategy to arrest autoimmune aggression in human T1D, benefiting from the co-delivery of tolerogenic signals and β-cell autoantigens.

## Materials and Methods

### Patients

Patients with T1D (*n* = 34) and control subjects (*n* = 24) were included in this study. All patients with diabetes fulfilled the classification criteria for T1D. Inclusion criteria were 18–55 years of age, a body mass index (BMI) between 18.5 and 30 kg/m^2^ and, for patients with T1D, an evolution of the disease longer than 6 months. Exclusion criteria were: being under immunosuppressive or anti-inflammatory treatment, or undergoing pregnancy or breastfeeding. For the RNA-sequencing (RNA-seq) experiment, we selected 8 patients of the 34 that participated in the study, but BMI was limited to a maximum of 24.9 kg/m^2^, duration of the disease was restricted to a maximum of 5 years (in order to minimize the effect that long-term hyperglycemia could have on genetic and/or epigenetic profiles) and the presence of other chronic diseases became an exclusion criterion. All study participants gave informed consent, and the study was approved by the Committee on the Ethics of Research of the Germans Trias i Pujol Research Institute and Hospital.

### Cell Separation and Generation of DCs

Peripheral blood mononuclear cells (PBMCs) were obtained from 50 ml blood samples of control subjects and patients with T1D by means of Ficoll Paque (GE Healthcare, Marlborough, MA, USA) density gradient centrifugation. Monocytes were further magnetically isolated using the EasySep Human CD14 Positive Selection Kit (STEMCELL Technologies, Vancouver, BC, Canada) following the manufacturer’s instructions. Once CD14 purity in the positively selected fraction was >70%, monocytes were cultured at a concentration of 10^6^ cells/ml in X-VIVO 15 media (Lonza, Basel, Switzerland), supplemented with 2% male AB human serum (Biowest, Nuaillé, France), 100 IU/ml penicillin (Normon SA, Madrid, Spain), 100 μg/ml streptomycin (Laboratorio Reig Jofré, Sant Joan Despí, Spain), and 1,000 IU/ml IL-4 and 1,000 IU/ml GM-CSF (Prospec, Rehovot, Israel) to obtain monocyte-derived DCs. After 6 days of culture, DC differentiation yield was assessed by CD11c-APC staining (Immunotools, Friesoythe, Germany) and cell viability was determined by annexin V-PE (Immunotools) and 7aad staining (BD Biosciences, San Jose, CA, USA) using flow cytometry (FACS Canto II, BD Biosciences). The negatively selected fraction of PBMCs was cryopreserved in Fetal Bovine Serum (ThermoFisher Scientific, Waltham, MA, USA) with 10% dimethylsulfoxide (Sigma-Aldrich, Saint Louis, MO, USA) and stored for later use.

### Peptide Selection and Preparation of Liposomes

Thinking in a future clinical application of liposomes, the two chains of insulin were selected to be encapsulated separately in order to avoid possible biological effects of insulin. A and B chains of insulin contain well-known β-cell specific target epitopes in human T1D (19). Peptide A corresponds to the whole human insulin A chain (21 aa, N-start-GIVEQCCTSICSLYQLENYCN-C-end), and peptide B is the whole human insulin B chain (30 aa, N-start-FVNQHLCGSHLVEALYLVCGERGFFYTPKT-C-end) (Genosphere Biotechnologies, Paris, France). Peptides were >95% pure and trifluoroacetic acid was removed. Liposomes consisted of 1,2-dioleoyl-sn-glycero-3-phospho-l-serine (sodium salt) (Lipoid, Steinhausen, Switzerland), 1,2-didodecanoyl-sn-glycero-3-phosphocholine (Lipoid), and cholesterol (Sigma-Aldrich). Liposomes were prepared using the thin film hydration method from a lipid mixture of 1,2-dioleoyl-sn-glycero-3-phospho-l-serine, 1,2-didodecanoyl-sn-glycero-3-phosphocholine and cholesterol at 1:1:1.33 molar ratio, respectively, as described (20). Liposomes without PS were generated as controls with 1,2-didodecanoyl-sn-glycero-3-phosphocholine and cholesterol at 1:1 molar ratio. All liposomes were produced under sterile conditions and at a final concentration of 30 mM. Lipids were dissolved in chloroform and the solvent was removed by evaporation under vacuum and nitrogen. The lipids were hydrated with the appropriate buffer (phosphate buffered saline or 0.5 mg/ml solution of peptide A or peptide B) and the liposomes obtained were homogenized to 1 μm by means of an extruder (Lipex Biomembranes Inc., Vancouver, BC, Canada). Peptide encapsulation efficiencies were calculated according to the equation: encapsulation efficiency (%) = [(C_peptide,total_-C_peptide,out_)/C_peptide,total_] ×100, where C_peptide,total_ is the initial peptide A or peptide B concentration and C_peptide,out_ is the concentration of non-encapsulated peptide. To measure the C_peptide,out_, liposome suspensions were centrifuged at 110,000 *g* at 10°C for 30 min. The concentration of non-encapsulated peptide was assessed in supernatants by PIERCE BCA protein assay kit (ThermoFisher Scientific). In addition to PS-rich liposomes loaded with insulin peptides [PSA-liposomes (*n* = 3) and PSB-liposomes (*n* = 3) encapsulating peptide A or peptide B, respectively], fluorescent-labeled liposomes with PS (empty fluorescent PS-liposomes, *n* = 4) and without PS (empty fluorescent PC-liposomes, *n* = 4) were also prepared using lipid-conjugated fluorescent dye Oregon Green 488 1,2-dihexadecanoyl-sn-glycero-3-phosphoethanolamine (Invitrogen, Carlsbad, CA, USA) and following the aforementioned methodology. Particle size distributions and stability—expressed as zeta potential (ζ)—were measured by dynamic light scattering using Malvern Zetasizer (Malvern Instruments, Malvern, UK) in undiluted samples. Liposome morphology and lamellarity were examined by cryogenic transmission electron microscopy (cryo-TEM) in a JEOL-JEM 1400 microscope (Jeol Ltd., Tokyo, Japan).

### Phagocytosis Assay

To assess whether DCs were able to phagocyte liposomes, DCs were co-cultured with 100 μM of empty fluorescent PS-liposomes (*n* = 5 for control subjects and *n* = 10 for patients with T1D) or empty fluorescent PC-liposomes (*n* = 6 for control subjects and *n* = 9 for patients with T1D) at 37°C from 5 min to 24 h. As control, the same assay was performed at 4°C to confirm that liposomes were captured by an active mechanism of phagocytosis. Cells were extensively washed in cold phosphate buffered saline to remove all liposomes attached to the cell membrane. Liposome uptake was determined by flow cytometry (FACSCanto II, BD Biosciences).

### Assessment of DCs Phenotype after Liposome Uptake

Although insulin chains were encapsulated separately, DCs were stimulated by a mixture of PSA-liposomes (50%) and PSB-liposomes (50%), in order to assess the effect of the whole insulin molecule as autoantigen. Thus, DCs from control subjects (*n* ≥ 5) and patients with T1D (*n* ≥ 8) were co-cultured with 1 mM of liposomes (PSAB-DCs) for 24 h in the presence of 20 μg/ml human insulin (Sigma-Aldrich), and their viability and phenotype were analyzed by flow cytometry (FACSCanto II, BD Biosciences). The sample number stated (*n*) is referred to the minimum number of control subjects and patients included in each experiment. As controls, DCs were either cultured with 20 μg/ml human insulin (Sigma-Aldrich) to obtain iDCs or adding a cytokine cocktail (CC) consisting of tumor necrosis factor (TNF)α (1,000 IU/ml, Immunotools), IL-1β (2,000 IU/ml, Immunotools) and Prostaglandin E_2_ (PGE_2_, 1 μM, Cayman Chemical, Ann Arbor, MI, USA) for 24 h to obtain mature DCs (mDC). Moreover, PSAB-DCs were cultured after phagocytosis with CC for 24 h in order to assess the response in front a pro-inflammatory stimulus (mPSAB-DCs). Phenotyping was performed as follows: DCs were stained with 7aad (BD Biosciences) and monoclonal antibodies to CD11c-APC, CD25-PE, CD86-FITC, HLA class I-FITC, HLA class II-FITC, CD14-PE and CD40-APC (Immunotools), CD36-APCCy7, TIM4-APC, αvβ5 integrin-PE, CD54-PECy7, TLR2-FITC, CXCR4-APCCy7, CCR2-APC, DC-SIGN-APC (Biolegend, San Diego, CA, USA) and CCR7-PECy7 (BD Biosciences). Corresponding fluorescence minus one staining was used as control. Data were analyzed using FlowJo software (Tree Star Inc., Ashland, OR, USA).

### T Cell Proliferation Assays

Autologous T lymphocyte proliferation (*n* = 12 for control subjects and *n* ≥ 12 for patients with T1D) was assessed by exposing PBMCs to the different conditions of DCs used in this study. Briefly, PBMCs from the same donor were thawed and stained with 0.31 μM CellTrace Violet (21) (ThermoFisher Scientific) according to manufacturer’s instructions. The PBMCs were then co-cultured with iDCs, PSAB-DCs, mPSAB-DCs or mDCs at a 10:1 ratio (10^5^ PBMCs:10^4^ DCs). For each donor, 10^5^ PBMCs were cultured in basal conditions as a negative control or with Phorbol 12-Myristate 13-Acetate (50 ng/ml, Sigma-Aldrich) and Ionomycin (500 ng/ml, Sigma-Aldrich) as a positive control. After 6 days of co-culture, proliferation was assessed in the different T cell subsets with CD3-PE, CD4-APC and CD8-FITC staining (Immunotools) by flow cytometry (FACS LSR Fortessa, BD Biosciences). Data were analyzed using FlowJo software (Tree Star Inc.).

### Cytokine Production

The Human Th1/Th2/Th17 kit (CBA system; BD Biosciences) was used to assess cytokine production. Culture supernatants from DCs and from T cell proliferation assays (*n* ≥ 3 for control subjects and *n* ≥ 3 for patients with T1D) were collected and frozen at −80°C until use. IL-2, IL-4, IL-6, IFN-γ, TNF, IL-17A, and IL-10 were measured. Data were analyzed using CBA software. The production of Human TGF-β1 by DCs after PSAB-liposome uptake was determined by ELISA (eBioscience, San Diego, CA, USA).

### RNA-Seq of DCs before and after Liposome Phagocytosis

Dendritic cells obtained from patients with T1D (*n* = 8) were cultured in basal conditions (iDCs) or with 1 mM of PSA-liposomes and PSB-liposomes (PSAB-DCs) at 37°C for 4 h. Cells were then harvested from culture wells using Accutase (eBioscience), and viability and DC purity were assessed with 7aad (BD Biosciences), annexin V-PE and CD11c-APC (Immunotools) staining by flow cytometry (FACS Canto II, BD Biosciences). Liposome capture control assays were performed for every sample (see above *Phagocytosis Assay* section) to confirm phagocytosis. Supernatant was removed and cell pellets were stored at −80°C until use. RNA was extracted using RNeasy Micro Kit (QIAGEN, Hilden, Germany) and following manufacturer’s instructions. RNA purity, integrity and concentration were determined by NanoDrop (ND-1000 Spectrophotometer, ThermoFisher Scientific) and 2100 Bioanalyzer (Agilent Technologies Inc., Santa Clara, CA, USA). Afterward, 1 μg of total RNA was used to prepare RNA libraries following the instructions of the NebNext Ultra Directional RNA Library Prep Kit (New England Biolabs, Ipswich, MA, USA). Library quality controls were assessed using a TapeStation 2200 (Agilent High Sensitivity Screen Tape) and a narrow distribution with a peak size of approximately 300 bp was observed in all cases. Libraries were quantified by qPCR using a QC KAPA kit (Hoffman-LaRoche, Basel, Switzerland) sequenced in a NextSeq 500 genetic analyzer (SBS-based sequencing technology, Illumina, San Diego, CA, USA) in a run of 2 × 75 cycles and a high output sequencing mode. Twenty million reads were obtained and analyzed for each sample. Fastq files coming from Illumina platform were merged and basic quality controls were performed with FASTQC and PRINSEQ tools. Paired-end (forward-reverse) sample merging was carried out with software CLCBio Genomics Workbench^®^ version 8.5 (22), following the RNA-seq analysis pipeline found in CLCBio manuals. Read alignment and mapping steps to only gene regions were performed using CLCBio software against the human genome (Homo sapiens GRCh38 assembly, at both gene- and transcript-level tracks). The same software, with default options, was used to normalize counts by applying standard “Reads Per Kilobase of transcript per Million reads mapped” method. The remaining steps of the analysis were carried out with scripts and pipelines implemented with R software (23). The selection of differentially expressed genes (DEGs) was performed using the linear model approach implemented in the limma Bioconductor package (24), with previous log2-transformation of the normalized data. Adjusted *p* values of ≤0.125, taking into account multiple testing with the False Discovery Rate method, were considered significant. Therefore, genes with a *p* value <0.0013 and Log_2_ of fold change >0.05 were considered upregulated, whereas those with Log_2_ of fold change <0.05 were considered downregulated. Experimental data have been uploaded into European Nucleotide Archive (EBI, https://www.ebi.ac.uk/ena; accession number: PRJEB22240). DEGs were categorized using Ingenuity Pathway Analysis Software (QIAGEN), Protein Analysis Through Evolutionary Relationships Classification System (25), REACTOME Pathway database (26) and Gene Ontology Biological Process database (27). Furthermore, R software (23) was used to generate a gene heatmap of DEGs.

### Quantitative RT-PCR

To validate transcriptome results, DCs obtained from patients with T1D (*n* ≥ 4) and control subjects (*n* ≥ 3) were cultured and pelleted in three conditions: iDCs, PSAB-DCs and mDCs. RNA was isolated using RNeasy Micro Kit (QIAGEN), and was reverse-transcribed with a High Capacity cDNA Reverse Transcription Kit (ThermoFisher Scientific). cDNA synthesis reactions were carried out using random hexamers (0.5 mg/ml, BioTools, Valle de Tobalina, Madrid, Spain) and reverse transcriptase Moloney-murine-Leukemia-virus (200 U/ml, Promega, Madison, WI, USA). Quantitative RT-PCR assays were performed with TaqMan universal assay (ThermoFisher Scientific) on a LightCycler^®^ 480 (Roche, Mannheim, Germany) using the following TaqMan assays: *CYTH4* (Hs01047905_m1), *GIMAP4* (Hs01032964_m1), *HPGD* (Hs00960590_m1), NFKB inhibitor alpha (*NFKBIA*) (Hs00153283_m1), *PLAUR* (Hs00958880_m1), *TNFAIP3* (Hs00234713_m1), tumor necrosis factor superfamily member 14 (*TNFSF14*) (Hs00542477_m1), and *VEGFA* (Hs00900055_m1). Relative quantification was performed by normalizing the expression for each gene of interest to that of the housekeeping gene *GAPDH* (Hs02758991_g1), as described in the 2^–ΔCt^ method (28).

### Statistical Analysis

The statistical analysis was performed using Prism 7.0 software (GraphPad software Inc., San Diego, CA, USA). Analysis of variance (ANOVA) was used for comparisons with several factors. For comparisons of unpaired data, a non-parametric Mann-Whitney test was used; for paired comparisons, a non-parametric Wilcoxon test was used. A *p* value ≤ 0.05 was considered significant.

## Results

### Patients with T1D and Control Subjects Display Similar Features

Thirty-four patients with T1D (50% female, 50% male) from the Germans Trias i Pujol Hospital and 24 control subjects (45.8% female, 54.2% male) met the inclusion and exclusion criteria and were included in the study (Table 1). Age of control subjects was 30.46 ± 8.18 years (mean ± SD), while that of patients with T1D was 32.54 ± 8.96 years; BMI was 23.90 ± 2.87 kg/m^2^ and 23.80 ± 3.11 kg/m^2^, respectively. Patients with T1D had been diagnosed at 20.79 ± 9.90 years, had a duration of disease of 11.75 ± 9.70 years, and a hemoglobin A1c level of 7.66 ± 1.26%. Control subjects did not significantly differ from patients with T1D in terms of age or BMI. Within the 34 patients, we selected 8 for the RNA-seq analysis, 50% female and 50% male, with a more stringent inclusion and exclusion criteria. Their age was 29.75 ± 5.85 years and their BMI was 22.50 ± 2.00 kg/m^2^. They had been diagnosed with T1D at 27.13 ± 7.43 years, had a duration of the disease of 2.50 ± 1.98 years and a hemoglobin A1c level of 7.36 ± 2.07%. Specific information on each subject can be found in Table 2.

**Table 1:** Data from the control subjects and patients with T1D recruited for the study.

	Control Subjects	Patients with T1D	*p* Value
*N*	24	34	—
Gender	11/24 (45.8%) Female	17/34 (50%) Female	—
	13/24 (54.2%) Male	17/34 (50%) Male	
Age (years)	30.46 ± 8.18	32.54 ± 8.96	0.4301
BMI (kg/m^2^)	23.90 ± 2.87	23.80 ± 3.11	0.9075
Age at T1D diagnosis (years)	NA	20.79 ± 9.90	—
Duration of T1D (years)	NA	11.75 ± 9.70	—
HbA1c (%)	NA	7.66 ± 1.26	—

*Data presented as mean ± SD; p value calculated from Mann–Whitney test*.

*T1D: type 1 diabetes; BMI, body mass index; NA, not applicable*.

**Table 2:** Data from the patients with T1D included in the RNA-seq experiment.

Patient number	Gender	Age (years)	BMI (kg/m^2^)	Age at T1D diagnosis (years)	Duration of T1D (years)	HbA1c (%)
1	Male	23	21.2	19	4	6.7
2	Female	28	24.4	23	5	6.5
3	Male	28	23.0	25	3	7.4
4	Female	33	21.4	33	0.5	5.9
5	Female	35	24.4	34	1	6.4
6	Female	32	18.6	31	0.5	5.9
7	Male	38	24.2	36	1	12.2
8	Male	21	23.0	16	5	7.9
Mean ± SD		29.75 ± 5.85	22.50 ± 2.00	27.13 ± 7.43	2.50 ± 1.98	7.36 ± 2.07

*Data presented as mean ± SD*.

*T1D, type 1 diabetes; BMI, body mass index*.

### DC Differentiation Efficiency Is Similar in Patients with T1D and Control Subjects

Monocytes were isolated magnetically from PBMCs. The yield of monocyte isolation—calculated as the percentage of the absolute number of CD14^+^ cells in the positively isolated fraction related to the absolute number of CD14^+^ cells in PBMCs—was 54.95 ± 24.97% (mean ± SD) for control subjects and 56.62 ± 18.12% for patients with T1D. The percentage of purity of CD14^+^ cells in the isolated fraction was 80.59 ± 10.18% for control subjects and 79.33 ± 7.56% for patients, and viability was 95.06 ± 4.14 and 94.92 ± 3.60%, respectively. The efficiency of differentiation to DCs at day 6 was 87.96 ± 6.61% for control subjects and 86.92 ± 6.86% for patients. No statistically significant differences were found when comparing these parameters between both groups. Data are detailed in Table 3.

**Table 3:** Data from monocyte’s isolation and dendritic cell differentiation.

	Yield (day 1, %)	Purity (day 1, %)	Viability (day 1, %)	Differentiation efficiency (day 6, %)
Control subjects	54.95 ± 24.97	80.59 ± 10.18	95.06 ± 4.14	87.96 ± 6.61
Patients	56.62 ± 18.12	79.33 ± 7.56	94.92 ± 3.60	86.92 ± 6.86
*p* Value	0.3789	0.2286	0.3664	0.5766

*Yield: % of the absolute number of CD14^+^ cells in the positively isolated fraction related to the absolute number of CD14^+^ cells in PBMCs. Purity: % of CD14^+^ cells in the isolated fraction. Viability: % of Annexin V^–^7aad^–^ cells. Differentiation efficiency: % of CD11c^+^ cells. Data presented as mean ± SD; p value calculated from Mann–Whitney test*.

*T1D, type 1 diabetes*.

### PS-Liposomes Show Multivesicular Vesicle Morphology and Encapsulate Insulin Peptides

**Figure 1:**
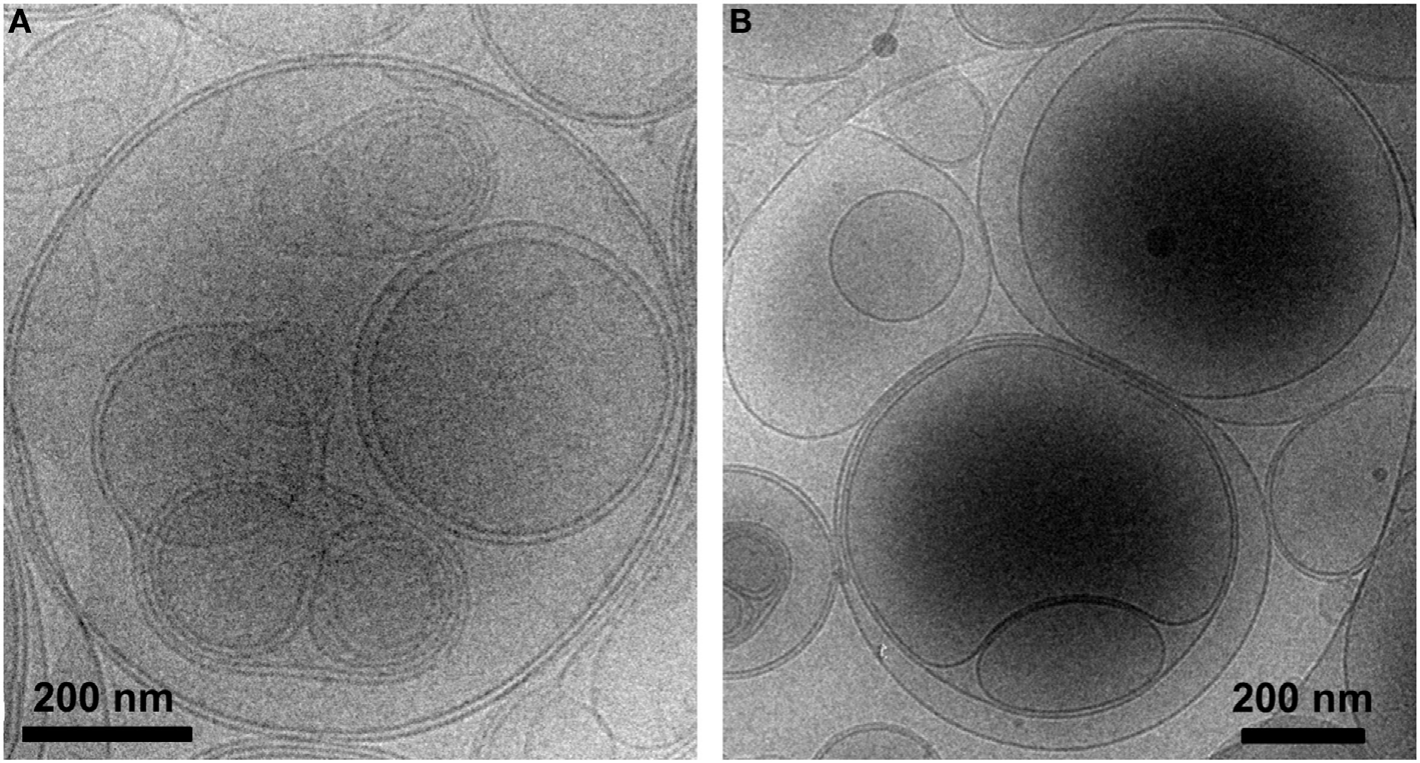
PS-liposomes display multivesicular and multilamellar morphology. Cryogenic transmission electron microscopy images of **(A)** PSA-liposomes (left) and **(B)** PSB-liposomes (right). Bar = 0.2 μm.

Liposomes were characterized in terms of diameter, polydispersity index (PdI), surface charge (ζ-potential) and efficiency of peptide encapsulation (Table 4). All liposomes had a final lipid concentration of 30 mM. All liposomes were large to guarantee efficient phagocytosis, displaying a diameter superior to 690 nm. The presence of PS molecules in liposomes was confirmed by the negative charge measured at the liposome surface by ζ-potential (−38 mV). Regarding specific features of PSA-liposomes (*n* = 3), the mean diameter was 690 ± 29 nm (mean ± SD), the PdI was 0.40 ± 0.28 and the ζ-potential was −38.57 ± 6.76 mV. The mean of peptide A (human insulin A chain) encapsulation efficiency was 39.74 ± 22.10%. As for PSB-liposomes (*n* = 3), they had a mean diameter of 788 ± 264 nm, the PdI was 0.52 ± 0.42 and the ζ-potential was −37.50 ± 7.16 mV, and the mean of peptide B (human insulin B chain) encapsulation efficiency was 93.19 ± 0.92%. Differences in peptide encapsulation efficiency (PSA *vs*. PSB) are due to amino acid composition and different solubility of insulin chains A (21 aa) and B (30 aa) in phosphate buffered saline media. B chain is more positively charged than A chain at neutral pH, resulting in a higher encapsulation efficiency in negatively charged liposomes. PSA-liposomes and PSB-liposomes presented multivesicular vesicle morphology when cryo-TEM analysis was performed (Figure 1).

**Table 4:** Features of the liposomes used in the study.

Liposome type	Diameter (nm)	Polydispersity index	ζ-potential (mV)	Encapsulation efficiency (%)
PSA-liposomes	690 ± 29	0.40 ± 0.28	−38.57 ± 6.76	39.74 ± 22.10
PSB-liposomes	788 ± 264	0.52 ± 0.42	−37.50 ± 7.16	93.19 ± 0.92
Fluorescent PS-liposomes	836 ± 217	0.32 ± 0.06	−38.90 ± 2.52	(empty)
Fluorescent PC-liposomes	1665 ± 488	0.32 ± 0.09	−7.60 ± 2.68	(empty)

*Data presented as mean ± SD*.

Fluorescent-labeled PS-liposomes (*n* = 4) showed a diameter of 836 ± 217 nm, a PdI of 0.32 ± 0.06 and a ζ-potential of −38.90 ± 2.52 mV. Their PS-free counterparts, fluorescent PC-liposomes (*n* = 4), had a diameter of 1665 ± 488 nm, a PdI of 0.32 ± 0.09 and a ζ-potential of −7.60 ± 2.68 mV (Table 4).

### Human DCs Display Optimal Kinetics of PS-Liposomes Phagocytosis without Affecting Viability

**Figure 2:**
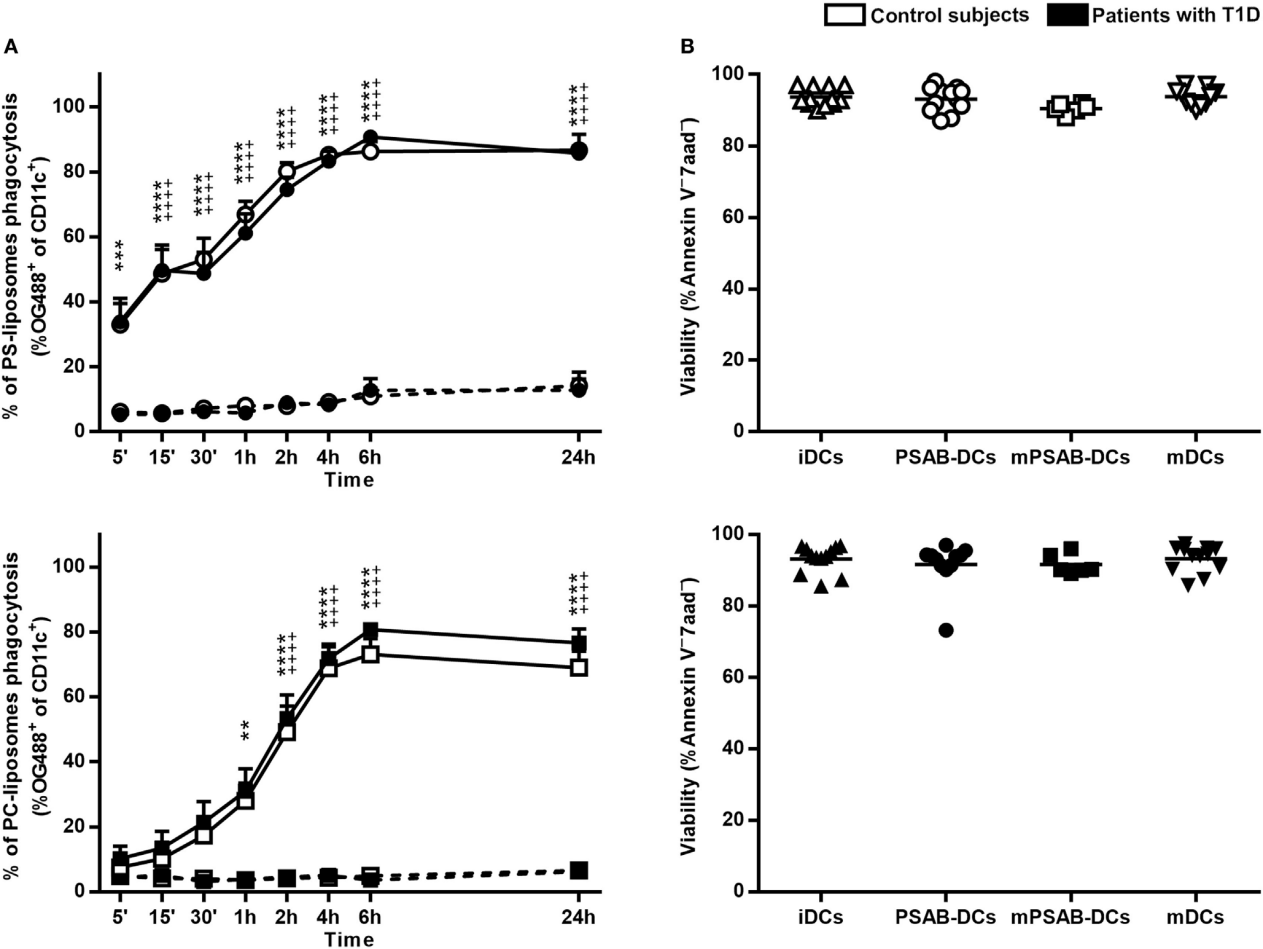
Liposomes are efficiently phagocyted by dendritic cells (DCs) and preserve a high viability. **(A)** Uptake of liposomes fluorescently labeled with lipid-conjugated fluorescent dye Oregon Green 488 1,2-dihexadecanoyl-sn-glycero-3-phosphoethanolamine by DCs. Upper panel: time course of the capture of fluorescently-labeled PS-liposomes by DCs obtained from control subjects (white circles, *n* = 5) and patients with type 1 diabetes (T1D) (black circles, *n* = 10) at 37°C (continuous line) and at 4°C (discontinuous line). Results are mean ± SEM. Comparisons between phagocytosis by control subjects DCs at 37 and 4°C showed significant differences [^++++^*p* < 0.0001, two-way analysis of variance (ANOVA)]; also, significant differences were found when comparing phagocytosis in patients with T1D at 37 and 4°C (***p < 0.001, ****p < 0.0001, Two-way ANOVA). No differences were found when comparing PS-liposomes uptake by DCs from control subjects and patients with T1D (Two-way ANOVA). Lower panel: time course of the capture of fluorescently-labeled PC-liposomes by DCs obtained from control subjects (white squares, *n* = 6) and patients with T1D (black squares, *n* = 9) at 37°C (continuous line) and at 4°C (discontinuous line). Results are mean ± SEM. Comparisons between phagocytosis by control subjects DCs at 37 and 4°C showed significant differences (^++++^*p* < 0.0001, Two-way ANOVA); also, significant differences were found when comparing phagocytosis in patients with T1D at 37 and 4°C (**p < 0.01, ****p < 0.0001, Two-way ANOVA). No differences were found when comparing PC-liposomes uptake by DCs from control subjects and patients with T1D (Two-way ANOVA). **(B)** Viability of DCs from control subjects (upper panel, white symbols, *n* ≥ 6) and patients with T1D (lower panel, black symbols, *n* ≥ 6) assessed by annexin V and 7aad staining. Triangles represent immature DCs (iDCs), circles represent iDCs cultured with PSA-liposomes and PSB-liposomes (PSAB-DCs), squares represent mature PSAB-DCs (mPSAB-DCs) and upside-down triangles represent mature DCs (mDCs). MDCs were induced by culture with cytokine cocktail.

A time course analysis was performed to determine PS-liposomes uptake kinetics (Figure 2A, upper left panel). The capture of empty fluorescent PS-liposomes by DCs was significantly higher at 37°C when compared to 4°C (*p* < 0.001), coming from either control subjects (*n* = 5) or patients (*n* = 10). This result is immunologically crucial and demonstrates that DCs engulf liposomes by an active mechanism of phagocytosis. PS-liposomes uptake kinetics were identical between control subjects and patients with T1D.

To indirectly assess the role of PS in phagocytosis, the same analysis was performed replacing PS-liposomes with PC-liposomes (Figure 2A, lower left panel). The percentages of empty PC-liposomes phagocytosis by DCs from control subjects (*n* = 6) and patients (*n* = 9) were significantly higher at 37°C when compared to 4°C starting at 2 h (*p* < 0.0001). The kinetics of the capture did not differ between control subjects and patients. When comparing uptake kinetics of PS- and PC-liposomes (Figure S1 in Supplementary Material), statistically significant differences were found, as expected. The presence of PS significantly accelerated phagocytosis in the first 2 h of co-culture (*p* < 0.05) both in control subjects and patients. In preliminary experiments, each type of liposome was tested in several sizes (diameter range 505–2,138 nm), and similar kinetics of capture were observed, independently of liposome size (Figure S2 in Supplementary Material), thus confirming that PS is the key factor in accelerating phagocytosis.

The viability of the different conditions of DCs (iDCs, PSAB-DCs, mPSAB-DCs, and mDCs) was assessed to determine liposome toxicity. The mean viability for each condition was always >90%, both in DCs obtained from control subjects (*n* ≥ 6) (Figure 2B, upper right panel), and patients with T1D (*n* ≥ 6) (Figure 2B, lower right panel).

### PS-Liposomes Uptake Regulates the Phenotypic Maturation of Human DCs

**Figure 3:**
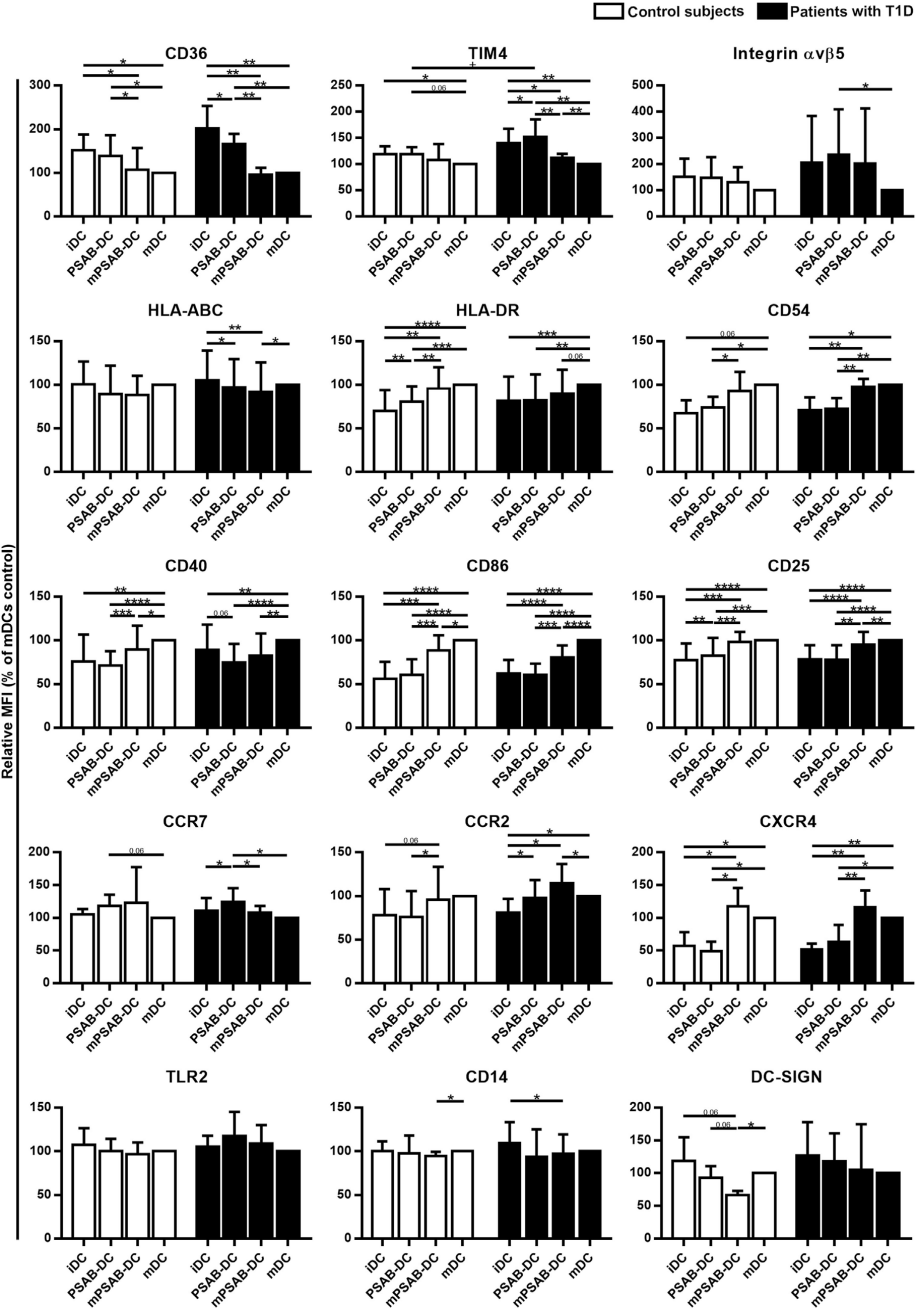
Capture of PSA-liposomes and PSB-liposomes regulates dendritic cell (DCs) phenotype. Relative CD36, TIM4, Integrin αvβ5, HLA-ABC, HLA-DR, CD54, CD40, CD86, CD25, CCR7, CCR2, CXCR4, TLR2, CD14, and DC-SIGN membrane expression in DCs obtained from control subjects (white bars, *n* ≥ 5) and patients with type 1 diabetes (T1D) (black bars, *n* ≥ 8). Bars represent immature DCs (iDCs), iDCs after the capture of PSA-liposomes and PSB-liposomes (PSAB-DCs), mature PSAB-DCs (mPSAB-DCs), or mature DCs (mDCs), 24 h after culture. MDCs were induced by culture with cytokine cocktail. Data presented as mean ± SD of relative Median of Fluorescence Intensity (MFI), this being MFI of each culture condition referred to their respective mDCs control. Significant differences were found when comparing culture conditions in the same group of subjects (*p ≤ 0.05, **p < 0.01, ***p < 0.001, ****p < 0.0001, Wilcoxon test), and when comparing the same culture conditions between patients with T1D and control subjects (^+^*p* < 0.05, Mann–Whitney test).

Changes in DCs phenotype were determined in control subjects (*n* ≥ 5) and patients with T1D (*n* ≥ 8). The membrane molecules assessed were: PS-receptors (CD36, TIM4, and αvβ5 integrin), antigen-presentation molecules (HLA-ABC and HLA-DR), adhesion molecules (CD54), costimulation molecules (CD40 and CD86), activation molecules (CD25), chemokine receptors (CCR7, CCR2, and CXCR4), and pattern recognition receptors (TLR2, CD14, and DC-SIGN). Figure 3 shows the relative Median of Fluorescence Intensity referred to mDCs.

PS-receptors CD36, TIM4 and αvβ5 integrin were expressed in iDCs. After liposome uptake, PSAB-DCs from patients decreased CD36 expression (*p* < 0.05) and upregulated TIM4 expression (*p* < 0.05) in comparison to iDCs, but PSAB-DCs presented a higher expression of CD36 and TIM4 than mDCs from control subjects and patients (*p* < 0.05). Moreover, PSAB-DCs from patients had increased levels of TIM4 in comparison to PSAB-DCs from control subjects (*p* < 0.05). The expression of αvβ5 integrin was higher in PSAB-DCs than in mDCs in patients (*p* < 0.05). As expected, CD36 and TIM4 were downmodulated in mDCs (*p* < 0.05), and αvβ5 integrin showed the same tendency.

Regarding HLA molecules, HLA-ABC was expressed similarly in iDCs and mDCs from both groups, and decreased in PSAB-DCs from patients after liposome capture (*p* < 0.05) —and control subjects displayed the same tendency. Concerning HLA-DR, iDCs showed a lower expression of this marker when compared to mDCs (*p* < 0.001). After liposome phagocytosis (PSAB-DCs), the low HLA-DR levels were preserved. As for the expression of adhesion molecule CD54, it was lower in iDCs from patients in comparison to mDCs (*p* < 0.05), and control subjects displayed the same tendency. After liposome uptake, no changes in CD54 expression were observed in PSAB-DCs when compared to iDCs, but mDCs displayed increased levels of CD54 in comparison to PSAB-DCs (*p* < 0.05). The expression of CD54 was higher in mPSAB-DCs exposed to a maturation stimulus despite the uptake of liposomes (*p* < 0.05).

Expression of costimulatory molecules CD40 and CD86 was lower in iDCs than in mDCs (*p* < 0.01). Liposome phagocytosis did not increase the expression of these molecules in PSAB-DCs. Moreover, PSAB-DCs presented lower levels of these markers when compared to mDCs (*p* < 0.0001), and even when exposed to pro-inflammatory stimulus (mPSAB-DCs) in comparison to mDCs (*p* < 0.05). Regarding the expression of activation marker CD25, it was lower in iDCs when compared to mDCs (*p* < 0.0001). Upregulation of CD25 was observed after liposome uptake in PSAB-DCs from control subjects (*p* < 0.01), but remained unaltered in patients. PSAB-DCs from both groups presented CD25 downmodulated when compared to mDCs (*p* < 0.001). Furthermore, DCs loaded with liposomes and exposed to pro-inflammatory stimulus (mPSAB-DCs) displayed lower levels of CD25 than mDCs in patients with T1D (*p* < 0.01).

Chemokine receptors CCR7 and CCR2 were expressed in iDCs. After liposome capture, the expression of both molecules increased in patients with T1D (*p* < 0.05). CCR7 was upregulated in PSAB-DCs when compared to mDCs in patients (*p* < 0.05), and control subjects displayed the same tendency. CXCR4, overexpressed in mDCs in comparison to iDCs (*p* < 0.05), was maintained low after liposome engulfment (PSAB-DCs). The expression of CXCR4 was higher in DCs exposed to a maturation stimulus despite the uptake of liposomes (mPSAB-DCs) (*p* < 0.05).

Pattern recognition receptors were assessed in DCs. TLR2 expression was similar in all experimental conditions, despite showing a tendency to increase after liposome phagocytosis (PSAB-DCs) in patients. CD14 was similarly expressed in iDCs and mDCs, but liposome uptake and maturation stimulus induced downregulation of this marker (*p* < 0.05). DC-SIGN, expressed in iDCs, displayed a tendency to be downmodulated after liposome capture (PSAB-DCs), especially in controls, which was more marked after a pro-inflammatory stimulus. Nonetheless, this marker showed a tendency to remain higher in PSAB-DCs than in mDCs in patients.

**Figure 4:**
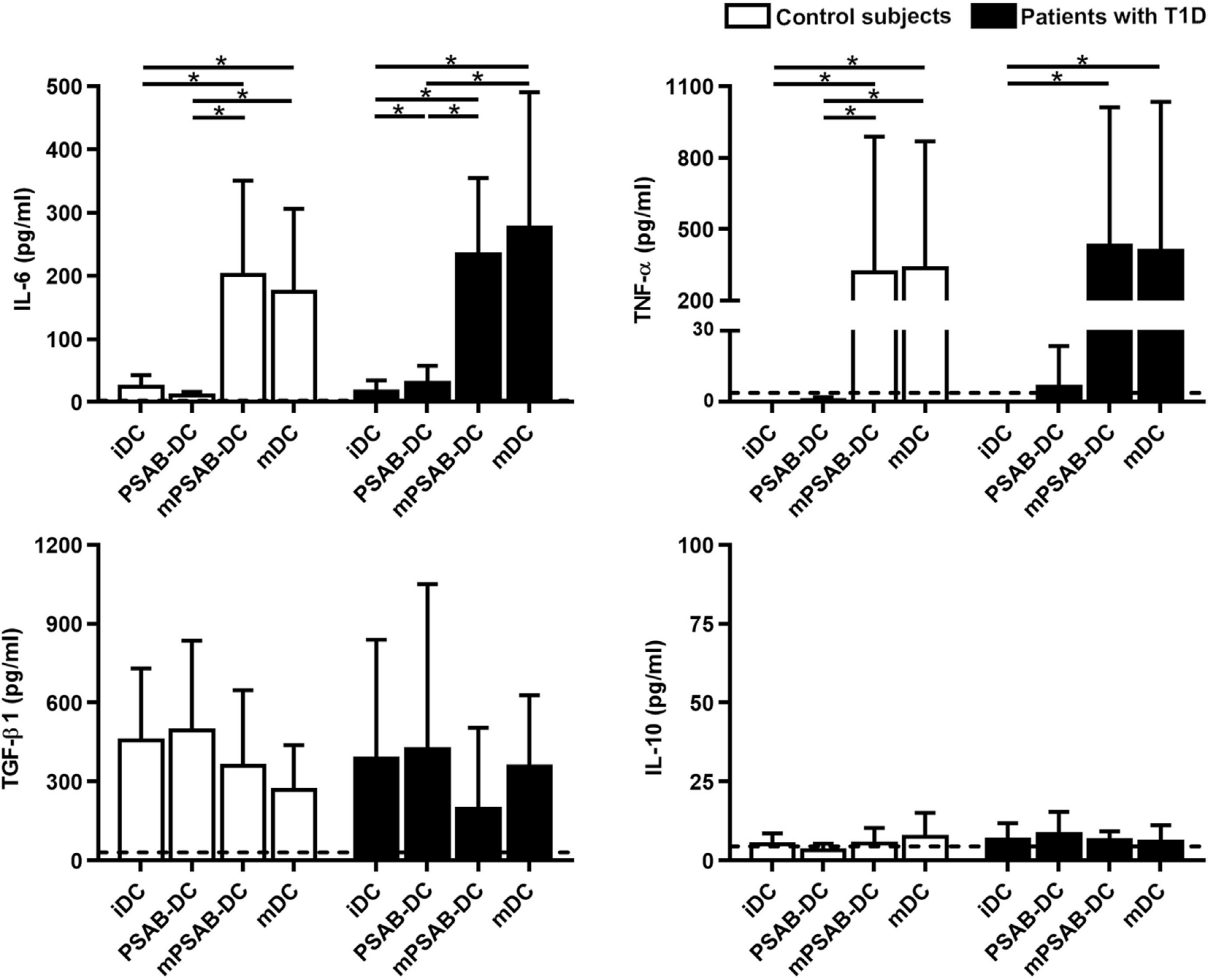
The uptake of PSAB-liposomes by dendritic cells (DCs) does not alter cytokine profile. Concentration (pg/ml) of IL-6, TNF-α, TGF-β1, and IL-10 secreted by DCs obtained from control subjects (white bars, *n* ≥ 3) and patients with type 1 diabetes (T1D) (black bars, *n* ≥ 3). Bars represent immature DCs (iDCs), iDCs after the capture of PSA-liposomes and PSB-liposomes (PSAB-DCs), mature PSAB-DCs (mPSAB-DCs), or mature DCs (mDCs). MDCs were induced by culture with cytokine cocktail. Data presented as mean ± SD. Significant differences were found when comparing the different conditions in the same group of subjects (*p < 0.05, Wilcoxon test), and differences were not found when comparing the same culture conditions between patients with T1D and control subjects (Mann–Whitney test).

In terms of cytokine secretion by DCs from patients (*n* ≥ 3) and control subjects (*n* ≥ 3) (Figure 4), IL-6 was released in low amounts after liposome phagocytosis and, as expected, its secretion increased after maturation stimulus. TNF-α was not increased after liposome uptake and its secretion increased in pro-inflammatory conditions. Liposome engulfment maintained a high profile of TGF-β1 secretion both in control subjects and patients, and tended to decrease in mPSAB-DCs and mDCs, although non-significant. Regarding IL-10 production, PSAB-DCs displayed a tendency to increase the secretion, although non-statistically significant, in patients with T1D. IL-2, IL-17A, and IFN-γ were not detected in any condition of the assay (data below the detection limit). IL-4 was not considered as it was used in culture media for DC differentiation.

### PS-Liposomes Uptake Impairs DCs Ability to Stimulate Autologous T Cell Proliferation

**Figure 5:**
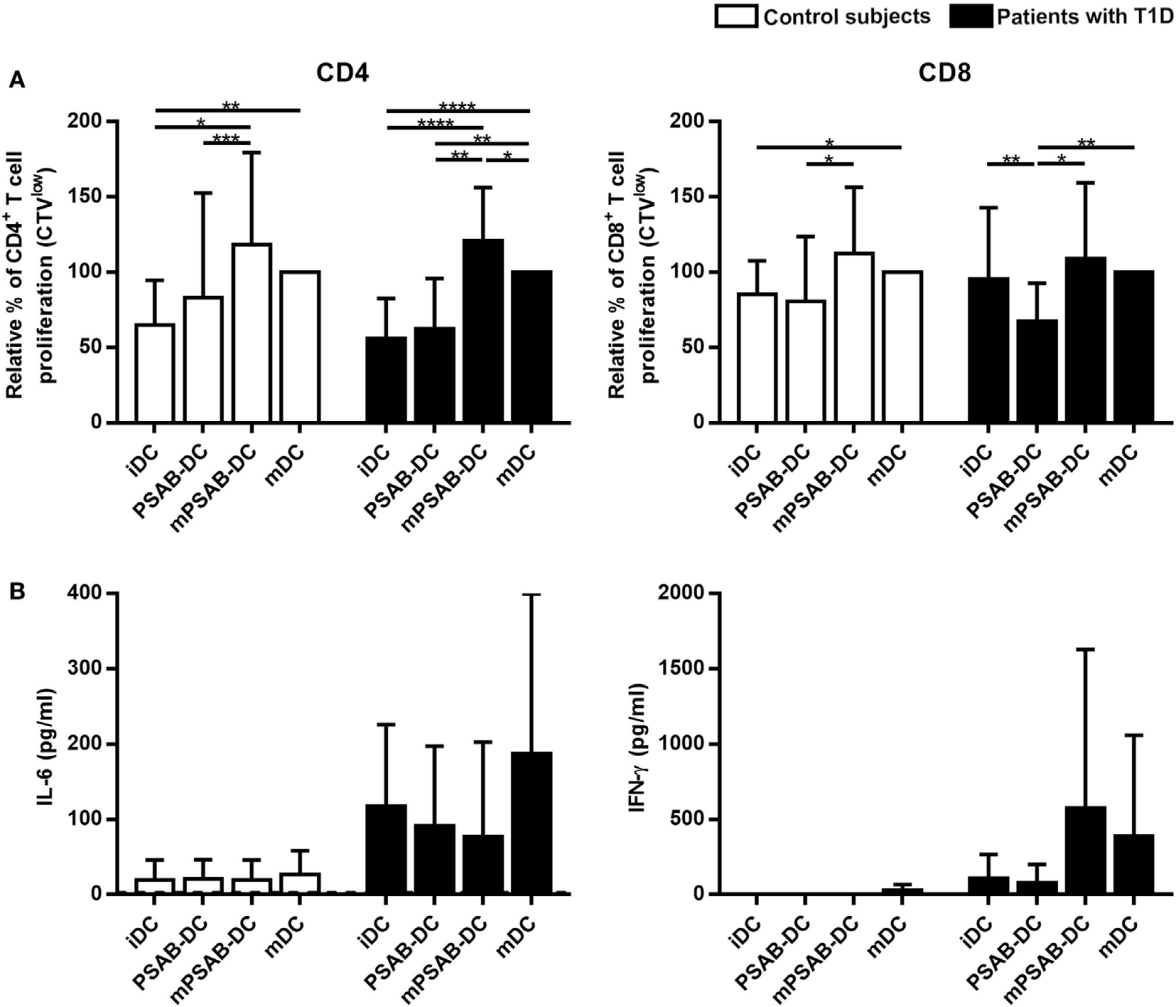
Capture of PSA-liposomes and PSB-liposomes affects dendritic cells (DCs) functionality. **(A)** Relative autologous proliferation of CD3^+^CD4^+^ and CD3^+^CD8^+^ subsets induced by DCs obtained from control subjects (white bars, *n* = 12) and patients with type 1 diabetes (T1D) (black bars, *n* ≥ 12). Autologous peripheral blood mononuclear cells were stained with CellTrace Violet (CTV) and co-cultured at 1:10 ratio for 6 days with each condition of DCs, and proliferation was measured as the percentage of CTV^low^ cells. Bars represent immature DCs (iDCs), iDCs after the capture of PSA-liposomes and PSB-liposomes (PSAB-DCs), mature PSAB-DCs (mPSAB-DC), or mature DCs (mDCs). MDCs were induced by culture with cytokine cocktail. Data presented as mean ± SD of relative proliferation induction, this being the percentage of CTV^low^ cells in each co-culture condition referred to that of their respective mDCs control. Significant differences were found when comparing culture conditions in the same group of subjects (*p ≤ 0.05, **p < 0.01, ***p < 0.001, ****p < 0.0001, Wilcoxon test), and differences were not found when comparing the same culture conditions between patients with T1D and control subjects (Mann-Whitney test). **(B)** IL-6 and IFN-γ secretion (pg/ml) assessed in supernatants of autologous proliferation co-culture with cells from control subjects (white bars, *n* ≥ 3) and patients with T1D (black bars, *n* ≥ 3). Bars represent immature DCs (iDCs), iDCs after the capture of PSA-liposomes and PSB-liposomes (PSAB-DCs), mature PSAB-DCs (mPSAB-DC), or mature DCs (mDCs). MDCs were induced by culture with cytokine cocktail. Data presented as mean ± SD. No differences were found when comparing the different conditions in the same group of subjects (Wilcoxon test), or when comparing the same culture conditions between patients with T1D and control subjects (Mann–Whitney test).

DCs derived from patients with T1D (*n* ≥ 12) and control subjects (*n* = 12) induced similar levels of autologous T cell proliferation (Figure 5). As expected, CD4^+^ T cell proliferation induced by mDCs was higher than proliferation induced by iDCs in both groups (*p* < 0.01). CD8^+^ T cell proliferation induced by mDCs was higher than proliferation induced by iDCs in control subjects (*p* < 0.05), but not in patients. Importantly, the capture of PSAB-liposomes by iDCs did not increase autologous CD4^+^ and CD8^+^ T cell proliferation, both in patients and control subjects. Moreover, a significant decrease of CD8^+^ T cell proliferation induced by PSAB-DCs from patients was observed after liposome capture, when compared to iDCs (*p* < 0.01). This effect was reverted after DCs maturation.

In terms of cytokine production, PBMCs co-cultured with PSAB-DCs displayed a cytokine profile (IL-6 and IFN-γ) similar to iDCs (Figure 5). Interestingly, PBMCs showed a tendency to increase IFN-γ and IL-6 secretion when co-cultured with mPSAB-DCs or mDCs, respectively, only in patients with T1D. IL-2, IL-4, IL-10, IL-17A, and TNF-α were not detected in any condition of the assay (data below the detection limit).

### Transcriptional Changes in DCs from Patients with T1D after PS-Liposomes Phagocytosis Point to an Immunoregulatory Prolife

RNA-seq analysis was performed in DCs from 8 patients with T1D (Table 2) in order to identify transcriptional changes after the capture of PS-liposomes. Phagocytosis was verified by flow cytometry using fluorescent liposomes. After 4 h of co-culture, 73.88 ± 11.57% (mean ± SD) of DCs were positive for fluorescent signal.

Integrity of the isolated RNA material was assessed for each sample, being optimal for RNA-seq experiment: RIN 9.0 ± 0.56 (mean ± SD). Bioinformatics analysis of the RNA-seq experiment revealed that only 233 of 22,711 genes detected were differentially expressed between iDCs and PSAB-DCs (*p* value < 0.0013, adjusted *p* value < 0.1254). Of these 233 genes, 203 (87.12%) were downregulated and the remaining 30 (12.88%) were upregulated, and 224 corresponded to protein-coding genes. Despite the heterogeneous basal transcriptomics of DCs from eight patients, gene expression was clearly altered toward a similar profile after PS-liposomes phagocytosis (Figure S3 in Supplementary Material).

We analyzed several categories and molecules related to DC function (Table S1 in Supplementary Material). DEGs were mainly related to metabolism, gene expression, immunoregulation, signal transduction, molecule transport, post-translational protein modification, cytokine signaling, cell cycle, vesicle-mediated processes, DNA replication and repair, antigen processing and presentation, apoptosis, and cytoskeleton organization (Table 5). Due to the immunotherapeutic potential of PS-liposomes, DEGs involved in tolerance were analyzed in detail. DEGs linked to the immune system were primarily downregulated and involved in antigen processing and presentation (*KBTBD6, BTK, CDC23, UBE2E3, CD1D*), regulation of the immune response (*DAPP1, GIMAP4, SLAMF6*) and cytokine signaling relevant in the interaction between T cells and DCs (*SOCS2, TNFRSF11A*). However, although very few genes were upregulated after PS-liposomes phagocytosis, these were related to the prevention of DC maturation [*TNFSF14, TNFAIP3, VEGFA, SHB*, leukocyte associated immunoglobulin-like receptor 1 (*LAIR1*), *NFKBIA*]. Also, genes related to apoptosis were downregulated in DCs after liposome uptake (*BLCAP, PMP22, LMNB1*).

**Table 5:** DEGs in dendritic cells (DCs) from patients with type 1 diabetes (T1D) after PSA-liposomes and PSB-liposomes phagocytosis.

Category	Number of DEGs	*P* value	Representative downregulated genes	Representative upregulated genes
Adhesion	3	≤0.001076	*SCYL3, MEGF9*	*IGSF9*

Antigen processing and presentation	7	≤0.001024	*KBTBD6, BTK, CDC23, UBE2E3, CD1D, CUL3, KIF11*	

Apoptosis	6	≤0.000593	*BLCAP, PMP22, LMNB1, CASP3, DCAF7, BCL2L1*	

Cell cycle	9	≤0.001162	*CSRP2BP, BUB1, MCPH1, CDK13, PCNA, MCM4, SMC2, NCAPG2, AURKA*	

Cytokine signaling	9	≤0.001191	*TRIM5, SOCS2, STX3, TNFRSF11A, NUP160*	*TNFSF14, VEGFA, TNF, IFNLR1*

Cytoskeleton organization	6	≤0.000897	*MAPRE2, RMDN1, CKAP2, MDM1, RCSD1, CDC42SE1*	

DNA replication and repair	8	≤0.000995	*WRNIP1, PAXIP1, MSH2, RAD51C, DCLRE1A, ALKBH1, PARG, MLH1*	

Gene expression	36	≤0.001293	*ZNF436, MYB, ZFP36L2, MIER3, ZBTB5, HHEX, GTF2B, DYRK2, NFIA, ZBTB39*	

Immunoregulation	25	≤0.001146	*GIMAP4, SLAMF6, DAPP1, MEF2C, BST1, PROS1, MNDA*	*TNFAIP3, PLAUR, NFKBIA*

Metabolism	43	≤0.001225	*C9orf64, HPGD, TIMMDC1, ICK, DDO, DCTD, CDYL2, GLRX, TPK1*	*MFSD2A*

Molecule transport	14	≤0.001252	*ERLIN1, SLC10A7, UNC50, ATP10D, SLC40A1, CLCN3, STIM2*	*SLC43A3, SLCO4A1, CLCN6*

Post-translational protein modification	13	≤0.001056	*FBXO36, NSMCE4A, VWA5A, FBXO25, DCAF12, CBX4, RMND5A, LNX2, BTBD3*	*PPME1*

Signal transduction	18	≤0.001298	*SNN, SKI, PAQR8, UBFD1, N4BP1, FZD5, NET1, ZBED3, FRAT2*	*SHB*

Vesicle-mediated processes	8	≤0.001208	*GOLPH3L, SEC22C, RAB32, EHBP1, KIF20B, SNX18*	*CYTH4, LDLR*

*DEGs, differentially expressed genes*.

**Figure 6:**
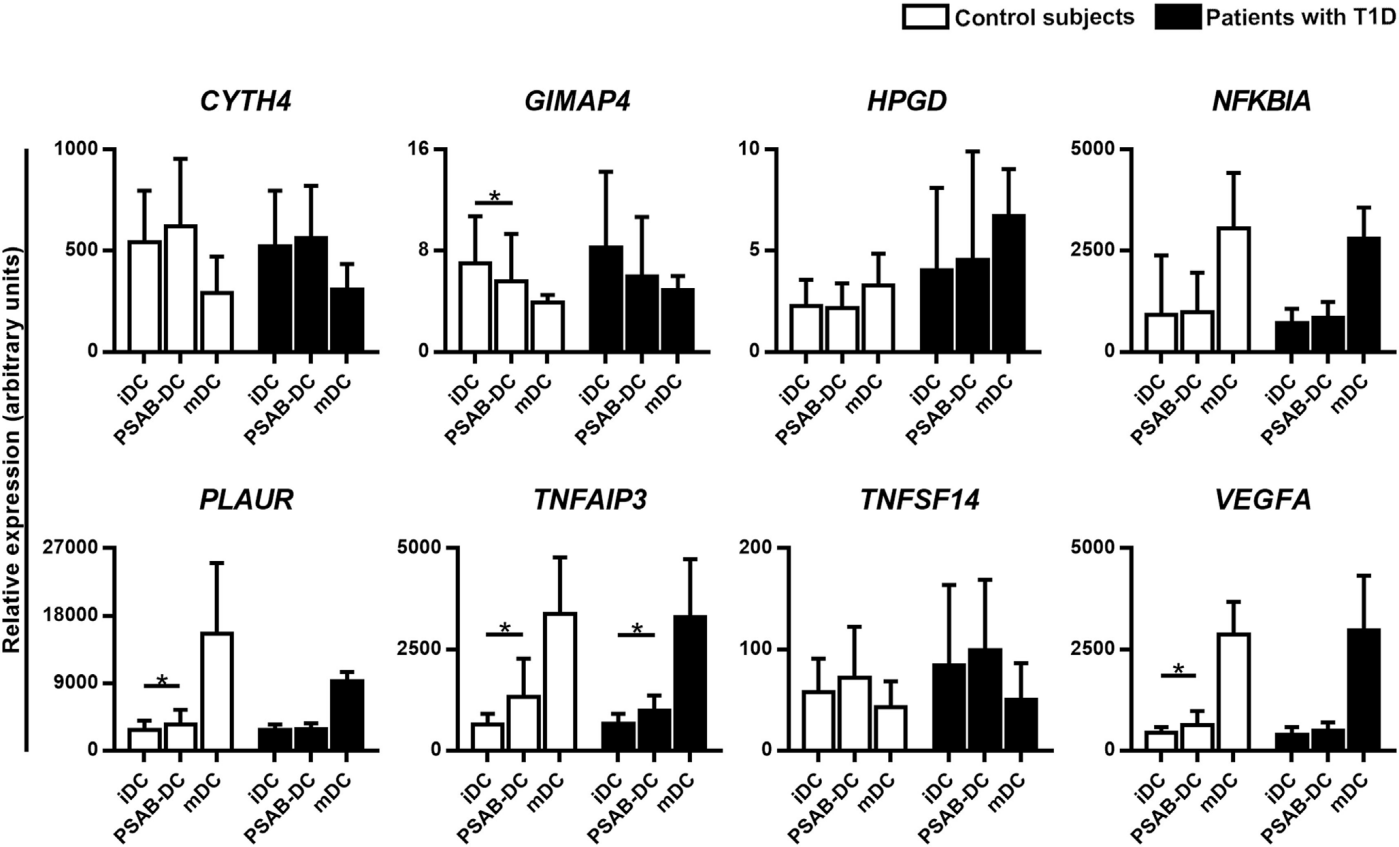
Quantitative RT-PCR validates the RNA-seq results. Relative gene expression of 8 selected targets in immature dendritic cells (iDCs), after phagocytosis of PSA-liposomes and PSB-liposomes (PSAB-DCs), and in mature dendritic cells (mDCs), analyzed by quantitative RT-PCR. Gene expression signals were normalized to *GAPDH*. Bars show the mean ± SD of gene expression in control subjects (white bars, *n* ≥ 3) and patients with type 1 diabetes (T1D) (black bars, *n* ≥ 4). Statistically significant differences were found when comparing the different conditions in the same group of subjects (*p < 0.05, Wilcoxon test), and differences were not found when comparing the same culture conditions between patients with T1D and control subjects (Mann–Whitney test).

Validation by qRT-PCR of the selected gene targets (*CYTH4, GIMAP4, HPGD, NFKBIA, PLAUR, TNFAIP3, TNFSF14*, and *VEGFA*) confirmed the RNA-seq results, when tested in DCs from 10 patients with T1D (Figure 6). As expected, gene expression analysis in DCs from 9 control subjects showed the same pattern. Regarding mDCs—from 4 patients with T1D and 3 control subjects—we observed a seemingly different gene expression pattern when compared to PSAB-DCs — and with iDCs. Genes upregulated by PS-liposomes, such as *CYTH4* and *TNFSF14*, tended to be downregulated in mDCs; other genes tended to be differentially expressed in mDCs (*NFKBIA, PLAUR, TNFAIP3, GIMAP4, VEGFA*, and *HPGD*) in comparison to the other conditions.

## Discussion

Apoptosis is a key factor in the maintenance of immunological tolerance. The uptake of apoptotic cells, through a process called efferocytosis, results in tolerogenic presentation of autoantigens inducing specific tolerance rather than autoimmunity (14). Therefore, the inherent immunomodulatory properties of apoptotic cells can be useful to design innovative immunotherapies. Based on this tolerogenic potential of efferocytosis, we generated liposomes that mimic apoptotic β-cells. At present, liposomes are clinically used mainly as vehicles for drugs (29–31), but they can be designed to modulate immune responses. This liposome-based immunotherapy resembles apoptotic cells and acts through the immunosuppressive signal of PS (32) and tolerogenic autoantigen presentation. These large PS-liposomes, after phagocytosis, are effective in restoring self-tolerance in experimental autoimmune diseases (16, 17) by their interaction with DCs and the arrest of the autoimmune reaction. To explore the clinical potential of this strategy, we have determined the effect of PS-liposomes loaded with human insulin peptides in DCs from patients with T1D. This effect has been assessed in several aspects: phagocytosis, phenotypic changes, effect on T cell proliferation and cytokine profile.

Regarding phagocytosis, lipid membrane composition is crucial for rapid engulfment by DCs, as demonstrated using PS- and PC-liposomes. As expected, the presence of PS accelerated phagocytosis of liposomes both in control subjects and patients with a dynamic typical of apoptotic cell clearance. PS-liposomes were more efficiently engulfed by DCs than the equivalent ones without PS in the first 2 h of co-culture, reaching plateau after 6 h. When encountering PS-liposomes, DCs are deceived into sensing that they are actual apoptotic cells that need to be rapidly efferocyted in order to avoid secondary necrosis that could contribute to autoimmunity (33, 34). Moreover, the preservation of DCs viability proved that PS-liposomes are not toxic, as reported for other types of liposomes (29–32).

The second aspect was the assessment of DCs phenotype. After liposome engulfment, PSAB-DCs maintained high levels of PS-receptors that mediate this uptake, when compared to mDCs, pointing to the preservation of phagocytosis ability in tolerogenic DCs (tolDCs). Interestingly, the upregulation of TIM4 expression observed in PSAB-DCs from patients might contribute to a positive feedback of phagocytosis. Upon maturation, PS-receptors were downmodulated correlating with the phagocytic capacity of mDCs, as described (35). The expression pattern of molecules involved in antigen presentation (HLA, costimulatory and adhesion molecules) in PSAB-DCs concurs with a tolerogenic function, both in patients and control subjects. The expression of CD25 activation marker, linked to DCs activation and autoimmunity (36, 37), confirmed the intermediate activation status of PSAB-DCs after phagocytosis. Also, the chemokine receptors expression pattern supports DCs ability to drive their migration to secondary lymphoid tissues (38, 39), and moreover, the high CCR7 expression is associated with induction of tolerance after efferocytosis (40). Additionally, the expression of pattern recognition receptors was not altered by liposomes, as described for human DCs (32). This phenotype is similar to the previously observed in mice (16, 17). Of note, RNA-seq analysis reinforces these results. Furthermore, upon liposome capture, the immunomodulatory cytokine TGFβ-1 was secreted, a reported effect driven by PS (34) that could suppress DC maturation and define the T cell response afterward. As expected, liposome capture did not induce IL-6 nor TNF-α secretion by DCs, but maturation did. Overall, the results point to the tolerogenic effect of these vesicles, which act on re-establishing self-tolerance. We observed minor phenotypic differences between DCs from patients and control subjects, which could be due to epigenetic changes caused by autoimmunity and metabolic dysregulation (41–43).

The third aspect was the analysis of autologous T cell proliferation induced by PSAB-DCs. In agreement with DCs phenotype, T cell proliferation induced by PSAB-DCs was similar or even lower than the induced by iDCs, both in patients and control subjects. Interestingly, in patients with T1D, there was a significant reduction in CD8^+^ T cell subset proliferation induced by tolDCs when compared to iDCs. This effect could be related to a reduction of the T cell cytotoxic activity, the most important effector response in human T1D (44, 45). In fact, after efferocytosis, DCs present apoptotic cell autoantigens to cognate T cells in the absence of costimulation, favoring tolerance to self (12, 13). It is reasonable to think that liposomes mimicking apoptotic cells will cause a similar effect, Additional studies using tetramers would be relevant to determine the antigen-specificity of the T cells involved in tolerance induction, even in pro-inflammatory conditions, in which T cells seem to proliferate more vigorously. Cytokines produced during the autologous T cell proliferation assay induced by PSAB-DCs discard a Th1 and Th17 profile, which could be detrimental in the induction of tolerance. In fact, IFN-γ, which is involved in a Th1 response, and IL-6 secretion, which partially contributes to induce Th17 response in T1D (46), remain poorly secreted in co-cultures of PBMCs with PSAB-DCs. Interestingly, higher amounts of IL-6 and IFN-γ tend to be produced by mDCs from patients with T1D when compared to controls. This feature could reflect the ongoing autoimmune reaction, present in peripheral blood from patients (47).

One of the obstacles of tolerogenic therapies in human disease is the heterogeneity of the *ex vivo*-generated tolDCs, which vary depending on the source, the manufacturing protocols, and the timespan of the experiment. Work is in progress to define and standardize a set of phenotypical and functional characteristics of tolDCs (48). To date, tolDCs are accepted as maturation-reluctant cells with low expression of antigen-presenting and costimulatory molecules and a tolerogenic-skewed cytokine profile (49). In this sense, one of the advantages of direct administration of the liposomes reported herein would be the generation of tolDCs *in vivo*, avoiding *ex vivo* cell manipulation. Our previous results in mice demonstrate this hypothesis (16, 17). However, a global picture of changes induced by PS-liposomes phagocytosis would grant a better understanding of tolerogenicity.

To fully characterize the immunomodulatory effects of liposomes, transcriptomic analysis was performed in DCs. Eight patients with a short T1D duration were selected in order to minimize the influence of long-term hyperglycemia on immune response, as reported (41–43). RNA-seq revealed a set of DEGs that avoid DCs maturation and contribute to tolerogenic antigen presentation. One of the most hyperexpressed genes was the vascular endothelial growth factor (VEGF) A (*VEGFA*), involved in cytokine signaling after efferocytosis (50), iDCs recruitment and maturation inhibition (51). VEGF increases the expression of the *TNFSF14* gene (52), also upregulated by PS-liposomes (53). In turn, TNFSF14 upregulates the production of TGF-β by phagocytes (54), and upon interacting with its ligand in T cells, TNFSF14 regulates T cell proliferation (55), inducing local immunosuppression (56). Supporting this fact, apoptotic cell clearance has been described to inhibit inflammation *via* TGF-β and VEGF production (34). Additionally, VEGFA enhances the expression of indoleamine 2,3-dioxygenase (*IDO*) (57), which in turn codifies for an immunomodulatory enzyme expressed in tolDCs (58). Moreover, the hematopoietically expressed homeobox (*HHEX*) gene, a repressor of VEGF signaling (59), is downregulated after PS-liposomes phagocytosis, whereas an inductor of VEGF expression, activating transcription factor 4 (*ATF4*) (60), is upregulated. Furthermore, the hyperexpressed SH2 domain containing adaptor protein B (*SHB*) gene codifies for a protein that regulates VEGF-dependent cellular migration (61), Th2 polarization and T regulatory cell induction (62). Other upregulated tolerogenic genes, such as the TNF alpha induced protein 3 (*TNFAIP3*) and the *LAIR1*, can inhibit DC maturation and their deficiency causes autoimmune and autoinflammatory diseases (63–66). In the same way, the hyperexpression of the *NFKBIA* gene would contribute to inhibit DC maturation and T cell activation (67, 68). Regarding cytokine signature, our results agree with those found in phenotypic and functional experiments. A relevant cytokine for tolerance induction is TGF-β1, secreted after efferocytosis (69). After PS-liposomes capture, DCs showed a biological increase of TGF-β1 transcription, although non-significant, probably due to the short timespan of the experiment (70). In fact, TGF-β1 was found in culture supernatants 24 h after PS-liposomes phagocytosis. Also, the immunoregulatory interferon lambda receptor 1 (*IFNLR1*) gene is one of the few overexpressed in DCs after PS-liposomes uptake. This receptor induces tolDCs that promote regulatory T cell expansion (71). Unexpectedly, the *TNF*-encoding gene was upregulated in DCs after liposome phagocytosis, and the same tendency was observed in protein secretion. Nevertheless, this behavior was very different to that observed in mDCs, which secreted higher amounts of TNF. The increase of TNF gene expression in our RNA-seq agrees with the upregulation of *TNFSF14* and *TNFAIP3* genes. Furthermore, a critical role for TNF has been reported in human tolDCs in the induction of antigen-specific regulatory T cells (72). Also, our previous results showed that murine tolDCs upregulated TNF-gene expression after efferocytosis (14). Overall, these results are consistent with the pleiotropic effects of TNF. Furthermore, in our previous research, PGE_2_ was found to be crucial in tolerance induced by PS-liposomes in mice (16). Strikingly, this pathway does not seem to be upregulated in human DCs, probably due to divergences between mice and men. Nevertheless, our data indirectly point to the involvement of the PGE_2_ pathway in human DCs: first, the downregulation of the hydroxyprostaglandin dehydrogenase 15-(NAD) (*HPGD*) gene, involved in PGE_2_ degradation, and second, a biological upregulation (although non-significant) of the peroxisome proliferator activated receptor gamma (PPARG), a gene induced by prostaglandins which is a negative regulator of pro-inflammatory cytokines (73). Furthermore, PGE_2_ has been described to stimulate the synthesis of VEGF (74). In summary, comparative transcriptome studies identify the whole molecular features of tolDCs rather than describe a simple state of maturation or lack thereof in terms of phenotype and function (75). Further studies are required to find a common signature of tolerogenicity, a fact hindered by individual differences of human DCs and the heterogeneous results obtained with different agents used to promote tolDCs. Our findings describe the specific gene signature of PS-liposomes-induced tolDCs. Their genomic program, which drives a different functionality than those of iDCs and mDCs, contributes to dissect the complexity of tolerance regulation.

Perhaps not so peculiarly, most of the alterations found in DCs after PS-liposomes capture are also physiopathological strategies used by tumor cells to escape immune surveillance. Small vesicles rich in PS are released by tumor cells and act as immunosuppressive agents to inhibit tumor antigen-specific T cells (76). Tumor cells can induce immunological tolerance using mechanisms characteristic of apoptotic cell clearance, and PS-liposomes seem to make use of the same pathways to achieve similar effects.

The use of PS-liposomes filled with autoantigens is an innovative strategy to arrest autoimmunity by restoring tolerance to self. As a whole, our results support the tolerogenic behavior of DCs, induced by the phagocytosis of PS-liposomes, and suggest that, in the context of autoimmunity, they could act silencing potential autoreactive T cells. This process could possibly be an active silencer, and not only a lack of maturation of DCs. In summary, here we unveil a picture of efferocytosis mimicry that leads to phenotypic and functional changes in human DCs, accountable for tolerance induction. The herein reported results reinforce the potential of this biocompatible immunotherapy to re-establish immunological tolerance, opening the door to new therapeutic approaches in the field of antigen-specific autoimmune disorders.

## Ethics Statement

This study was carried out after the approval and in strict accordance with the recommendations of the guidelines of Germans Trias i Pujol Ethical Committee. All subjects gave written informed consent in accordance with the Declaration of Helsinki.

## Author Contributions

SR-F, IP-A, MC-S, FV, JV, and MV-P designed the study and interpreted the data; SR-F, DM, JV, and MV-P wrote the manuscript; SR-F, IP-A, DP-B, MC-S, SG-J, and AV performed the experiments; IP-A, SR-F, DM, and MV-P supervised the experiments; SR-F, EA, FV, and MV-P selected the patients and supervised the collection of blood samples; and SR-F, IP-A, FB, and AS analyzed the data. All authors revised the work and gave final approval of the version to be published.

## Conflict of Interest Statement

IP, MC, JV, DM, and MV are inventors in a patent (WO2015107140) that describes the use of autoantigen-encapsulating liposomes for the prevention or treatment of autoimmune disorders.

## Abbreviations

DC, dendritic cell; PS, phosphatidylserine; T1D, type 1 diabetes.
