# Supplementary material for: Phosphatidylserine-Liposomes Promote Tolerogenic Features on Dendritic Cells in Human Type 1 Diabetes by Apoptotic Mimicry
Source: Front Immunol. 2018 Feb 14;9:253. doi: 10.3389/fimmu.2018.00253 (PMC5817077; doi:10.3389/fimmu.2018.00253)
Supplement: Supplementary file 1 [file Table_1.PDF]

Table S1. Differentially expressed genes (DEGs) in dendritic cells (DCs) from patients with type 1 diabetes (T1D) after phagocytosis of PSA-liposomes and PSB-liposomes, according to categories based on biological processes.

Gene Symbol	Gene ID	Gene Name	Log_2 FC	P value
**ADHESION**				
SCYL3	57147	SCY1 like pseudokinase 3	-0.08	0.000396
MEGF9	1955	Multiple EGF like domains 9	-0.52	0.000656
IGSF9	57549	Immunoglobulin superfamily member 9	0.18	0.001076
**ANTIGEN PROCESSING AND PRESENTATION**				
KBTBD6	89890	Kelch repeat and BTB domain containing 6	-0.15	0.000002
BTK	695	Bruton tyrosine kinase	-0.16	0.000291
CDC23	8697	Cell division cycle 23	-0.22	0.000397
UBE2E3	10477	Ubiquitin conjugating enzyme E2 E3	-0.19	0.000417
CD1D	912	CD1d molecule	-0.61	0.000595
CUL3	8452	Cullin 3	-0.22	0.000731
KIF11	3832	Kinesin family member 11	-0.12	0.001024
**APOPTOSIS**				
BLCAP	10904	Bladder cancer associated protein	-0.17	0.000175
PMP22	5376	Peripheral myelin protein 22	-0.33	0.000251
LMNB1	4001	Lamin B1	-0.26	0.000417
CASP3	836	Caspase 3	-0.37	0.000449
DCAF7	10238	DDB1 and CUL4 associated factor 7	-0.26	0.000488
BCL2L11	10018	BCL2 like 11	-0.41	0.000593
**CELL CYCLE**				
CSRP2BP	57325	Cysteine-rich protein 2-binding protein	-0.16	0.000111
BUB1	699	BUB1 mitotic checkpoint serine/threonine kinase	-0.06	0.000269
MCPH1	79648	Microcephalin 1	-0.10	0.000337
CDK13	8621	Cyclin dependent kinase 13	-0.15	0.000424
PCNA	5111	Proliferating cell nuclear antigen	-0.41	0.000691
MCM4	4173	Minichromosome maintenance complex component 4	-0.16	0.000925
SMC2	10592	Structural maintenance of chromosomes 2	-0.25	0.000946
NCAPG2	54892	Non-SMC condensin II complex subunit G2	-0.08	0.001090
AURKA	6790	Aurora kinase A	-0.26	0.001162
**CYTOKINE SIGNALING**				
TNFSF14	8740	TNF superfamily member 14	0.61	0.000003
VEGFA	7422	Vascular endothelial growth factor A	0.30	0.000025
TRIM5	85363	Tripartite motif containing 5	-0.23	0.000047
SOCS2	8835	Suppressor of cytokine signaling 2	-0.12	0.000054
STX3	6809	Syntaxin 3	-0.28	0.000121
TNF	7124	Tumor necrosis factor	0.48	0.000175
IFNLR1	163702	Interferon lambda receptor 1	0.16	0.000193
TNFRSF11A	8792	Tumor necrosis factor receptor superfamily member 11A	-0.17	0.000677
NUP160	23279	Nucleoporin 160	-0.15	0.001191
**CYTOSKELETON ORGANIZATION**				
MAPRE2	10982	Microtubule associated protein RP/EB family member 2	-0.16	0.000163

RMDN1	51115	Regulator of microtubule dynamics 1	-0.15	0.000238
CKAP2	26586	Cytoskeleton associated protein 2	-0.31	0.000320
MDM1	56890	Mdm1 nuclear protein	-0.13	0.000504
RCSD1	92241	RCSD domain containing 1	-0.34	0.000801
CDC42SE1	56882	CDC42 small effector 1	-0.21	0.000897
**DNA REPLICATION AND REPAIR**				
WRNIP1	56897	Werner helicase interacting protein 1	-0.12	0.000017
PAXIP1	22976	PAX interacting protein 1	-0.09	0.000028
MSH2	4436	DNA mismatch repair protein Msh2	-0.25	0.000188
RAD51C	5889	RAD51 paralog C	-0.09	0.000374
DCLRE1A	9937	DNA cross-link repair 1A	-0.15	0.000586
ALKBH1	8846	ALKB homolog 1, histone H2A dioxygenase	-0.11	0.000912
PARG	8505	Poly(ADP-ribose) glycohydrolase	-0.16	0.000985
MLH1	4292	MutL homolog 1	-0.16	0.000995
**EXTRACELLULAR MATRIX ORGANIZATION**				
MFAP1	4236	Microfibril associated protein 1	-0.19	0.000130
SPARC	6678	Secreted protein acidic and cysteine rich	-0.24	0.001197
**GENE EXPRESSION**				
ZNF436	80818	Zinc finger protein 436	-0.16	0.000001
MYB	4602	MYB proto-oncogene, transcription factor	-0.06	0.000007
ZFP36L2	678	ZFP36 ring finger protein like 2	-0.51	0.000017
MIER3	166968	MIER family member 3	-0.13	0.000034
ZBTB5	9925	Zinc finger and BTB domain containing 5	-0.13	0.000091
HHEX	3087	Hematopoietically expressed homeobox	-0.40	0.000113
GTF2B	2959	General transcription factor IIB	-0.27	0.000117
DYRK2	8445	Dual specificity tyrosine phosphorylation regulated kinase 2	-0.18	0.000219
NFIA	4774	Nuclear factor I A	-0.07	0.000238
ZBTB39	9880	Zinc finger and BTB domain containing 39	-0.09	0.000260
NIF3L1	60491	NGG1 interacting factor 3 like 1	-0.17	0.000366
ZBTB10	65986	Zinc finger and BTB domain containing 10	-0.16	0.000424
PRMT6	55170	Protein arginine methyltransferase 6	-0.27	0.000430
TFB2M	64216	Transcription factor B2, mitochondrial	-0.18	0.000439
ASCC1	51008	Activating signal cointegrator 1 complex subunit 1	-0.13	0.000448
SAP30L	79685	Histone deacetylase complex subunit SAP30L	-0.17	0.000478
H2AFV	94239	H2A histone family member V	-0.19	0.000517
SMARCA2	6595	SWI/SNF related, matrix associated, actin dependent regulator of chromatin, subfamily a, member 2	-0.32	0.000538
SRPRB	58477	SRP receptor beta subunit	-0.18	0.000574
KLF10	7071	Kruppel like factor 10	-0.26	0.000576
BRD3	8019	Bromodomain containing 3	-0.25	0.000713
PPARA	5465	Peroxisome proliferator activated receptor alpha	-0.14	0.000750
INTS7	25896	Integrator complex subunit 7	-0.14	0.000763
SESN3	143686	Sestrin 3	-0.27	0.000795
ZHX1	11244	Zinc fingers and homeoboxes 1	-0.28	0.000816
PARN	5073	Poly(A)-specific ribonuclease	-0.24	0.000862
SNUPN	10073	Snurportin 1	-0.11	0.000893
GTF2E1	2960	General transcription factor IIE subunit 1	-0.19	0.000962

LRRC32	2615	Leucine rich repeat containing 32	0.43	0.000983
RBM17	84991	RNA binding motif protein 17	-0.16	0.000983
NCBP1	4686	Nuclear cap binding protein subunit 1	-0.19	0.001140
ZNF717	100131827	Zinc finger protein 717	-0.07	0.001234
ZNF318	24149	Zinc finger protein 318	-0.16	0.001235
FLI1	2313	Fli-1 proto-oncogene, ETS transcription factor	-0.28	0.001262
OVOL1	5017	Ovo like transcriptional repressor 1	0.22	0.001286
ZNF107	51427	Zinc finger protein 107	-0.08	0.001293
**IMMUNOREGULATION**				
TNFAIP3	7128	TNF alpha induced protein 3	0.53	0.000004
GIMAP4	55303	GTPase, IMAP family member 4	-0.51	0.000008
SLAMF6	114836	SLAM family member 6	-0.12	0.000016
DAPP1	27071	Dual adaptor of phosphotyrosine and 3-phosphoinositides 1	-0.27	0.000025
MEF2C	4208	Myocyte enhancer factor 2C	-0.09	0.000045
PLAUR	5329	Plasminogen activator, urokinase receptor	0.32	0.000123
BST1	683	Bone marrow stromal cell antigen 1	-0.17	0.000160
NFKBIA	4792	NFKB inhibitor alpha	0.54	0.000181
PROS1	5627	Protein S (alpha)	-0.14	0.000317
MNDA	4332	Myeloid cell nuclear differentiation antigen	-0.42	0.000405
MLEC	9761	Malectin	-0.26	0.000457
METTL7A	25840	Methyltransferase like 7A	-0.45	0.000602
TLR5	7100	Toll-like receptor 5	-0.18	0.000637
MAP2K3	5606	Mitogen-activated protein kinase kinase 3	0.33	0.000702
LRMP	4033	Lymphoid restricted membrane protein	-0.25	0.000720
SRC	6714	SRC proto-oncogene, non-receptor tyrosine kinase	0.33	0.000721
DUSP4	1846	Dual specificity phosphatase 4	0.12	0.000786
SNAP29	9342	Synaptosome associated protein 29	-0.17	0.000790
ZC3HAV1	56829	Zinc finger CCCH-type containing, antiviral 1	-0.22	0.000833
IFI16	3428	Interferon gamma inducible protein 16	-0.38	0.000834
SH3BP2	6452	SH3 domain binding protein 2	0.22	0.000869
GAPT	202309	GRB2 binding adaptor protein, transmembrane	-0.16	0.000888
ALDOC	230	Aldolase, fructose-bisphosphate C	0.16	0.000975
LAIR1	3903	Leukocyte associated immunoglobulin like receptor 1	0.12	0.001022
ATF4	468	Activating transcription factor 4	0.17	0.001146
**METABOLISM**				
C9orf64	84267	Chromosome 9 open reading frame 64	-0.22	0.000037
HPGD	3248	Hydroxyprostaglandin dehydrogenase 15-(NAD)	-0.09	0.000053
TIMMDC1	51300	Translocase of inner mitochondrial membrane domain containing 1	-0.15	0.000123
ICK	22858	Intestinal cell kinase	-0.17	0.000213
DDO	8528	D-aspartate oxidase	-0.20	0.000255
DCTD	1635	dCMP deaminase	-0.15	0.000257
CDYL2	124359	Chromodomain Y like 2	-0.12	0.000312
GLRX	2745	Glutaredoxin	-0.15	0.000313
MFSD2A	84879	Major facilitator superfamily domain containing 2A	0.54	0.000317
TPK1	27010	Thiamin pyrophosphokinase 1	-0.17	0.000325
PDPR	55066	Pyruvate dehydrogenase phosphatase regulatory subunit	-0.14	0.000338
SPTLC2	9517	Serine palmitoyltransferase long chain base subunit 2	-0.31	0.000344

PDK4	5166	Pyruvate dehydrogenase kinase 4	-0.54	0.000400
HCCS	3052	Holocytochrome c synthase	-0.22	0.000431
SPTSSA	171546	Serine palmitoyltransferase small subunit A	-0.33	0.000463
FAR2	55711	Fatty acyl-CoA reductase 2	-0.32	0.000470
HERPUD1	9709	Homocysteine inducible ER protein with ubiquitin like domain 1	-0.27	0.000472
CLYBL	171425	Citrate lyase beta like	-0.06	0.000506
KLF4	9314	Kruppel like factor 4	-0.30	0.000506
INSIG1	3638	Insulin induced gene 1	1.13	0.000533
ALDH3A2	224	Aldehyde dehydrogenase 3 family member A2	-0.28	0.000534
EPM2A	7957	EPM2A, laforin glucan phosphatase	-0.09	0.000562
INPP1	3628	Inositol polyphosphate-1-phosphatase	0.10	0.000587
EXTL2	2135	Exostosin like glycosyltransferase 2	-0.22	0.000598
CLPX	10845	Caseinolytic mitochondrial matrix peptidase chaperone subunit	-0.26	0.000635
ACOX1	51	Acyl-CoA oxidase 1	-0.20	0.000644
ALDH5A1	7915	Aldehyde dehydrogenase 5 family member A1	-0.17	0.000705
THRAP3	9967	Thyroid hormone receptor associated protein 3	-0.19	0.000723
DIS3L	115752	DIS3 like exosome 3'-5' exoribonuclease	-0.14	0.000792
TMLHE	55217	Trimethyllysine hydroxylase, epsilon	-0.18	0.000814
ENO2	2026	Enolase 2	0.38	0.000828
ERI2	112479	ERI1 exoribonuclease family member 2	-0.22	0.000845
SCO1	6341	SCO1, cytochrome c oxidase assembly protein	-0.13	0.000859
EPRS	2058	Glutamyl-prolyl-tRNA synthetase	-0.25	0.000874
GLRX2	51022	Glutaredoxin 2	-0.23	0.000911
PARP9	83666	Poly(ADP-ribose) polymerase family member 9	-0.21	0.000996
GNPDA2	132789	Glucosamine-6-phosphate deaminase 2	-0.09	0.001026
GTF2I	2969	General transcription factor IIi	-0.17	0.001057
DNAJC16	23341	DnaJ heat shock protein family (Hsp40) member C16	-0.17	0.001065
STARD7	56910	StAR related lipid transfer domain containing 7	-0.34	0.001099
FIG4	9896	FIG4 phosphoinositide 5-phosphatase	-0.28	0.001105
LIAS	11019	Lipoic acid synthetase	-0.18	0.001124
ACP6	51205	Acid phosphatase 6, lysophosphatidic	-0.06	0.001225
**MOLECULE TRANSPORT**				
ERLIN1	10613	ER lipid raft associated 1	-0.25	0.000016
SLC10A7	84068	Solute carrier family 10 member 7	-0.17	0.000169
UNC50	25972	UNC-50 inner nuclear membrane RNA binding protein	-0.26	0.000354
ATP10D	57205	TPase phospholipid transporting 10D (putative)	-0.16	0.000491
SLC40A1	30061	Solute carrier family 40 member 1	-0.23	0.000640
SLC43A3	29015	Solute carrier family 43 member 3	0.23	0.000646
CLCN3	1182	Chloride voltage-gated channel 3	-0.37	0.000672
SLCO4A1	28231	Solute carrier organic anion transporter family member 4A1	0.56	0.000724
CLCN6	1185	Chloride voltage-gated channel 6	0.20	0.000767
STIM2	57620	Stromal interaction molecule 2	-0.22	0.000993
ATP2A2	488	ATPase sarcoplasmic/endoplasmic reticulum Ca2+ transporting 2	-0.20	0.001067
SLC30A1	7779	Solute carrier family 30 member 1	-0.52	0.001132
TMEM184C	55751	Transmembrane protein 184C	-0.19	0.001167
SLC1A3	6507	Solute carrier family 1 member 3	0.20	0.001252

**NON-CODING PROTEIN GENES**				
AC009506.1	643072	Uncharacterized LOC643072	-0.21	0.000014
AC093323.3	93622	Morf4 Family Associated Protein 1 Like 1 Pseudogene (LOC93622)	-0.14	0.000740
CARD8-AS1	100505812	CARD8 antisense RNA 1	-0.44	0.000078
DHRS4-AS1	55449	DHRS4 antisense RNA 1	-0.17	0.000620
GGTA1P	2681	Glycoprotein, alpha-galactosyltransferase 1 pseudogene	-0.25	0.000928
MIR762HG	101928736	MIR762 host gene	-0.08	0.000437
RP11-465B22.3	100288175	Uncharacterized LOC100288175	0.21	0.000555
RP11-506M13.3	101929506	Uncharacterized LOC101929506	-0.09	0.000694
ZNF542P	147947	Zinc finger protein 542, pseudogene	-0.12	0.000113
**OTHER**				
ABHD17B	51104	Abhydrolase domain containing 17B	-0.26	0.000158
C11orf54	28970	Chromosome 11 open reading frame 54	-0.19	0.000568
C5orf30	90355	Chromosome 5 open reading frame 30	-0.14	0.000179
FAM118B	79607	Family with sequence similarity 118 member B	-0.22	0.000029
HERPUD2	64224	HERPUD family member 2	-0.23	0.000162
SPIN3	169981	Spindlin family member 3	-0.11	0.001076
TRAF3IP1	26146	TRAF3 interacting protein 1	-0.12	0.000580
**POST-TRANSLATIONAL PROTEIN MODIFICATION**				
PPME1	51400	Protein phosphatase methylesterase 1	0.13	0.000062
FBXO36	130888	F-box protein 36	-0.11	0.000192
NSMCE4A	54780	NSE4 homolog A, SMC5-SMC6 complex component	-0.18	0.000419
VWA5A	4013	Von Willebrand factor A domain containing 5A	-0.21	0.000616
FBXO25	26260	F-box protein 25	-0.08	0.000669
DCAF12	25853	DDB1 and CUL4 associated factor 12	-0.27	0.000672
CBX4	8535	Chromobox 4	-0.21	0.000704
RMND5A	64795	Required for meiotic nuclear division 5 homolog A	-0.34	0.000710
LNX2	222484	Ligand of numb-protein X 2	-0.15	0.000821
BTBD3	22903	BTB domain containing 3	-0.21	0.000926
TMEM5	10329	Transmembrane protein 5	-0.11	0.000987
PREP	5550	Prolyl endopeptidase	-0.18	0.001025
KCTD2	23510	Potassium channel tetramerization domain containing 2	-0.25	0.001056
**SIGNAL TRANSDUCTION**				
SNN	8303	Stannin	-0.43	0.000007
SHB	6461	SH2 domain containing adaptor protein B	0.31	0.000026
SKI	6497	SKI proto-oncogene	-0.26	0.000069
PAQR8	85315	Progestin and adipoQ receptor family member 8	-0.38	0.000160
UBFD1	56061	Ubiquitin family domain containing 1	-0.10	0.000209
N4BP1	9683	NEDD4-binding protein 1	-0.20	0.000221
FZD5	7855	Frizzled class receptor 5	-0.07	0.000329
NET1	10276	Neuroepithelial cell transforming 1	-0.23	0.000369
ZBED3	84327	Zinc finger BED-type containing 3	-0.21	0.000615
FRAT2	23401	FRAT2, WNT signaling pathway regulator	-0.41	0.000675
PEX11B	8799	Peroxisomal biogenesis factor 11 beta	-0.19	0.000726
TFDP2	7029	Transcription factor Dp-2	-0.12	0.000787

DACT1	51339	Dishevelled binding antagonist of beta catenin 1	-0.16	0.000831
RGS18	64407	Regulator of G protein signaling 18	-0.38	0.001044
MAP4K4	9448	Mitogen-activated protein kinase kinase kinase kinase 4	-0.12	0.001103
ARHGAP25	9938	Rho GTPase activating protein 25	-0.17	0.001144
AHR	196	Aryl hydrocarbon receptor	-0.57	0.001222
ABHD2	11057	Abhydrolase domain containing 2	-0.45	0.001298
**UNCHARACTERIZED**				
C21orf91	54149	Chromosome 21 open reading frame 91	-0.21	0.000813
EVI2B	2124	Ecotropic viral integration site 2B	-0.60	0.000405
FAM102B	284611	Family with sequence similarity 102 member B	-0.26	0.000813
FAM105A	54491	Family with sequence similarity 105 member A	-0.33	0.001104
FAM120C	54954	Family with sequence similarity 120C	-0.07	0.000614
FAM217B	63939	Family with sequence similarity 217 member B	-0.19	0.000825
PGBD2	267002	PiggyBac transposable element derived 2	-0.12	0.000096
TMEM248	55069	Transmembrane protein 248	-0.16	0.000560
TRANK1	9881	Tetratricopeptide repeat and ankyrin repeat containing 1	-0.14	0.000779
YPEL2	388403	Yippee like 2	-0.31	0.000874
**VESICLE-MEDIATED PROCESSES**				
CYTH4	27128	Cytohesin 4	0.31	0.000013
GOLPH3L	55204	Golgi phosphoprotein 3 like	-0.28	0.000084
SEC22C	9117	SEC22 homolog C, vesicle trafficking protein	-0.14	0.000341
LDLR	3949	Low density lipoprotein receptor	0.80	0.000672
RAB32	10981	RAB32, member RAS oncogene family	-0.17	0.000762
EHBP1	23301	EH domain binding protein 1	-0.08	0.000840
KIF20B	9585	Kinesin family member 20B	-0.15	0.000851
SNX18	112574	Sorting nexin 18	-0.37	0.001208
