# Supplementary material for: Phosphatidylserine-Liposomes Promote Tolerogenic Features on Dendritic Cells in Human Type 1 Diabetes by Apoptotic Mimicry
Source: Front Immunol. 2018 Feb 14;9:253. doi: 10.3389/fimmu.2018.00253 (PMC5817077; doi:10.3389/fimmu.2018.00253)
Supplement: Supplementary file 2 [file Image_1.PDF]

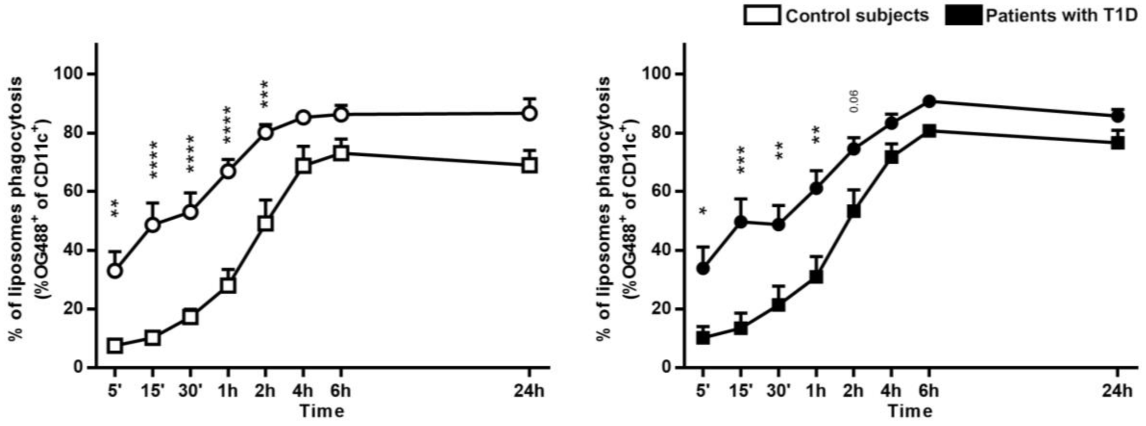

Figure S1. Comparison between time courses of PS-liposomes and PC-liposomes capture by dendritic cells (DCs) from control subjects and patients with type 1 diabetes (T1D). **Left panel**: time course of the capture of fluorescently-labeled PS-liposomes (circles) and PC-liposomes (squares), performed by DCs from control subjects (white symbols, n=5) at 37 ºC. Results are mean±standard error of the mean (SEM). Comparison between PS-liposomes' and PC-liposomes phagocytosis at different time points showed significant differences (**p<0.01, ***p<0.001, ****p<0.0001, Two-way ANOVA). **Right panel**: time course of the capture of fluorescently-labeled PS-liposomes (circles) and PC-liposomes (squares), performed by DCs from patients with T1D (n=10) at 37 ºC. Results are mean±SEM. Comparison between PS-liposomes' and PC-liposomes phagocytosis at different time points showed significant differences (*p<0.05, **p<0.01, ***p<0.001, Two-way ANOVA).
