# Supplementary material for: Phosphatidylserine-Liposomes Promote Tolerogenic Features on Dendritic Cells in Human Type 1 Diabetes by Apoptotic Mimicry
Source: Front Immunol. 2018 Feb 14;9:253. doi: 10.3389/fimmu.2018.00253 (PMC5817077; doi:10.3389/fimmu.2018.00253)
Supplement: Supplementary file 3 [file Image_2.PDF]

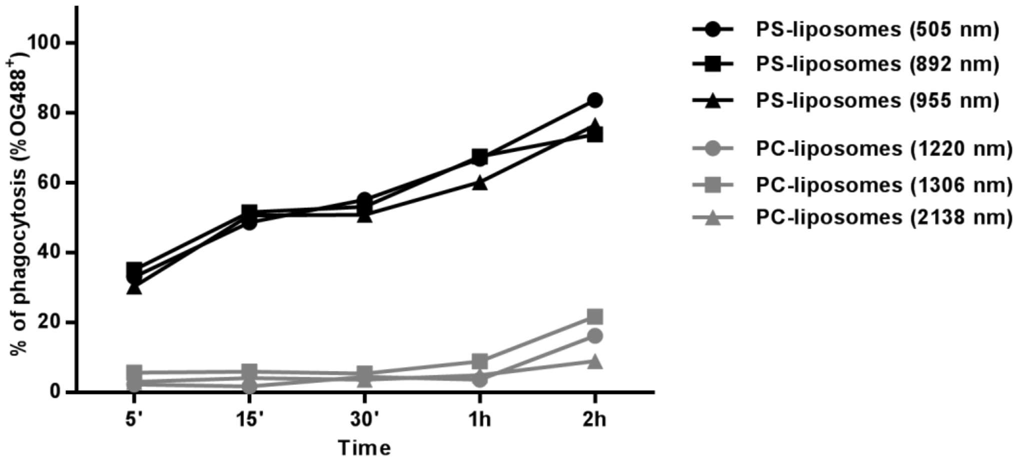

Figure S2. Time course of the capture of liposomes with different diameter size. Uptake of liposomes fluorescently-labeled with lipid-conjugated fluorescent dye Oregon Green 488 1,2-ihexadecanoyl-sn-glycero-3-phosphoethanolamine, performed by human dendritic cells at 37 ^{0}C. Black lines indicate empty fluorescent PS-rich liposomes (PS-liposomes) with 505 nm (circles), 892 nm (squares) and 955 nm (triangles) of diameter; grey lines show empty fluorescent PS-free liposomes (PC-liposomes) with 1220 nm (circles), 1306 nm (squares) and 2138 nm (triangles) of diameter.
