# Supplementary material for: Phosphatidylserine-Liposomes Promote Tolerogenic Features on Dendritic Cells in Human Type 1 Diabetes by Apoptotic Mimicry
Source: Front Immunol. 2018 Feb 14;9:253. doi: 10.3389/fimmu.2018.00253 (PMC5817077; doi:10.3389/fimmu.2018.00253)
Supplement: Supplementary file 4 [file Image_3.PDF]

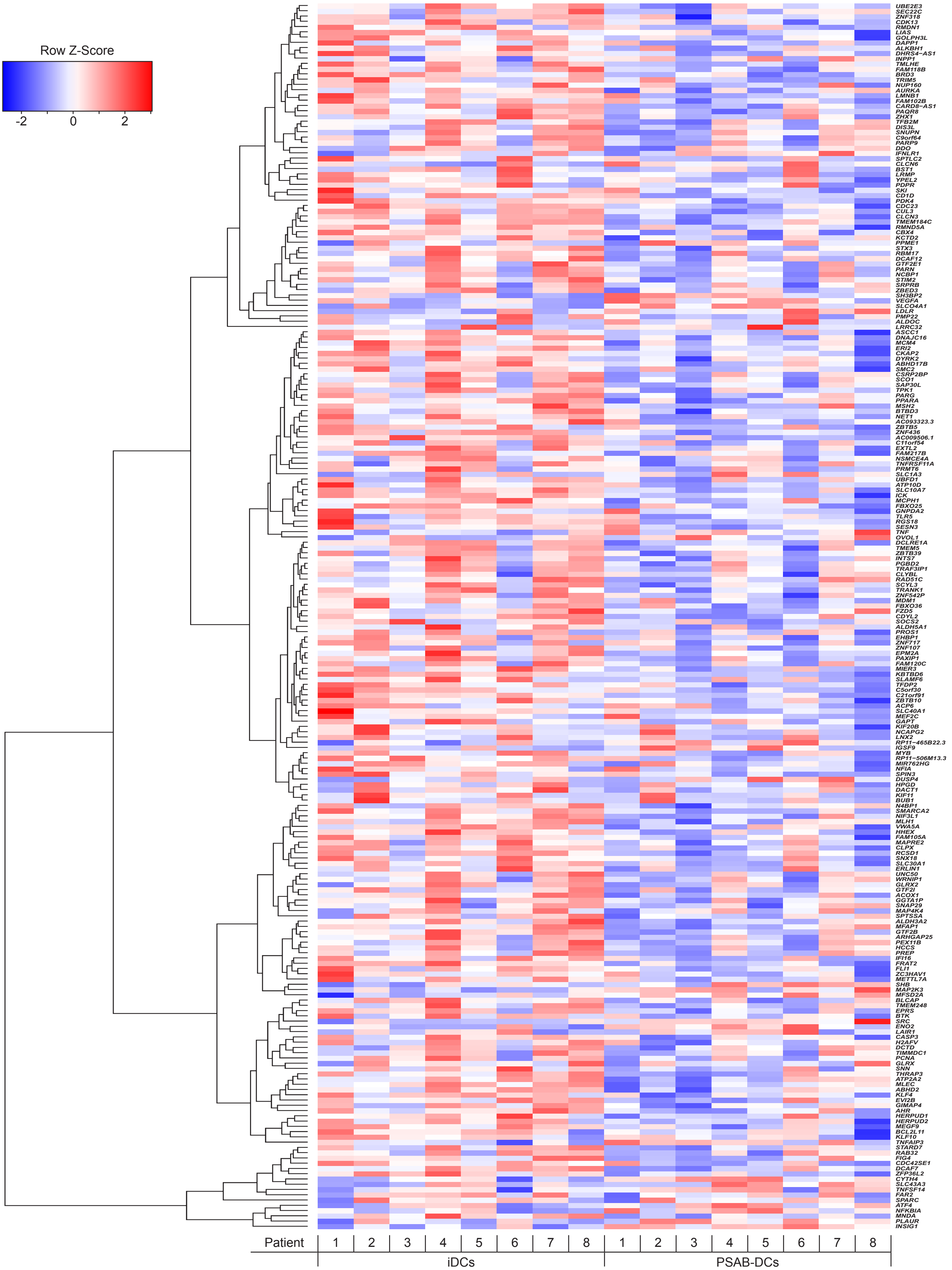

Figure S3. Heatmap of the differentially expressed genes (DEGs) in dendritic cells (DCs) from patients with type 1 diabetes (T1D) after phagocytosis of PSA-liposomes and PSB-liposomes. A heatmap of the 233 genes considered as DEGs (p value <0.0013, adjusted p value 0.1254, and Log_2 of fold change >0.05 or <–0.05) resulting from the RNA-seq analysis. The first 8 columns represent individual samples of immature DCs (iDCs) obtained from 8 patients with T1D, whereas the remaining 8 columns represent DCs after the phagocytosis of PSA-liposomes and PSB-liposomes (PSAB-DCs) from the same patients. The rows show specific genes of interest. The Row Z-score of each gene measures deviation from its mean, and it is depicted by color intensity, with blue indicating downregulated and red indicating upregulated genes.
